# Transient Receptor Potential Vanilloid-1 Channels Facilitate Axonal Degeneration of Corneal Sensory Nerves in Dry Eye

**DOI:** 10.1016/j.ajpath.2024.01.015

**Published:** 2024-02-05

**Authors:** Manuela Pizzano, Alexia Vereertbrugghen, Agostina Cernutto, Florencia Sabbione, Irene A. Keitelman, Carolina M. Shiromizu, Douglas Vera Aguilar, Federico Fuentes, Mirta N. Giordano, Analía S. Trevani, Jeremías G. Galletti

**Affiliations:** * the Innate Immunity Laboratory, Institute of Experimental Medicine ( CONICET/ National Academy of Medicine of Buenos Aires), Buenos Aires, Argentina; † the Confocal Microscopy Unit, Institute of Experimental Medicine ( CONICET/ National Academy of Medicine of Buenos Aires), Buenos Aires, Argentina

## Abstract

Corneal nerve impairment contributes significantly to dry eye disease (DED) symptoms and is thought to be secondary to corneal epithelial damage. Transient receptor potential vanilloid-1 (TRPV1) channels abound in corneal nerve fibers and respond to inflammation-derived ligands, which increase in DED. TRPV1 overactivation promotes axonal degeneration *in vitro*, but whether it participates in DED-associated corneal nerve dysfunction is unknown. To explore this, DED was surgically induced in wild-type and TRPV1-knockout mice, which developed comparable corneal epithelial damage and reduced tear secretion. However, corneal mechanosensitivity decreased progressively only in wild-type DED mice. Sensitivity to capsaicin (TRPV1 agonist) increased in wild-type DED mice, and consistently, only this strain displayed DED-induced pain signs. Wild-type DED mice exhibited nerve degeneration throughout the corneal epithelium, whereas TRPV1-knockout DED mice only developed a reduction in the most superficial nerve endings that failed to propagate to the deeper subbasal corneal nerves. Pharmacologic TRPV1 blockade reproduced these findings in wild-type DED mice, whereas CD4^+^ T cells from both strains were equally pathogenic when transferred, ruling out a T-cell–mediated effect of TRPV1 deficiency. These data show that ocular desiccation triggers superficial corneal nerve damage in DED, but proximal propagation of axonal degeneration requires TRPV1 expression. Local inflammation sensitized TRPV1 channels, which increased ocular pain. Thus, ocular TRPV1 overactivation drives DED-associated corneal nerve impairment.

The cornea is the eye’s most powerful refractive lens, but it can only fulfill its role when it is kept wet, smooth, and transparent.^1^ At the same time, the cornea is continuously exposed to rapidly changing environmental conditions that pose a threat to its integrity, and with it, to proper sight. To cope with this challenge, the cornea is sheltered within the ocular surface^2–4^ and is endowed with abundant innervation that reaches peak density in its anterior-most layer, the corneal epithelium.^5,6^ The intraepithelial corneal nerve fibers detect fluctuations in moisture, temperature, and osmolarity and provide sensory input to the protective neural mechanisms that control blinking and tearing.^7^ Thus, corneal innervation plays an important role in corneal function and protection.^7^

Dry eye disease (DED) is a multifactorial and increasingly prevalent ocular surface disorder typified by a dysfunctional tear film, which ultimately leads to ocular discomfort and pain, corneal damage, and impaired sight.^8^ Initially, desiccation causes tear hyperosmolarity and epithelial cell injury, both of which elicit an inflammatory response that aggravates tissue damage, thus generating a self-perpetuating vicious cycle.^9^ In addition to the ocular surface epithelium, corneal nerves are also affected in DED.^7^ Human studies show that there is a decrease in corneal nerve density, reduced fiber thickness, increased beading, tortuosity and sprouting, and signs of abnormal regeneration,^10–19^ and animal models of the disease reproduce these findings.^20–25^ Corneal nerve dysfunction in and of itself also reinforces DED.^7,12,26^ But contrasting the current understanding of how the corneal epithelium is affected by the disease,^27–31^ it remains to be determined if other pathophysiological mechanisms specifically target corneal nerves in DED.

Corneal innervation comprises a heterogeneous population of fibers that can be broadly classified by their functional and biochemical properties into three types: mechanonociceptors, cold thermoreceptors, and polymodal nociceptors.^7,26,32,33^ The first two respond to pressure or temperature changes, whereas polymodal nociceptors react to diverse noxious stimuli. Corneal polymodal nociceptor fibers typically express transient receptor potential vanilloid-1 (TRPV1) channels,^7,12,34^ which are activated by heat, low pH, hyperosmolarity, and various endogenous ligands that are increased in the context of inflammation and cell damage.^35^ Thus, TRPV1 signaling is critical to nociception and pain perception.^36^ In the skin, TRPV1 activation leads to proinflammatory neuropeptide release from local nerve fibers in a process known as cutaneous neurogenic inflammation.^37^ Consistently, TRPV1 activation in the ocular surface also promotes substance P release with the subsequent neurogenic proinflammatory effects.^38^ On the other hand, TRPV1 overstimulation leads to neuronal and nonneuronal cell death *in vitro*,^39–43^ and corneal TRPV1 activity reportedly increases in the context of DED.^31,44–47^ However, whether TRPV1 channels play a role in the development of DED-associated corneal nerve dysfunction remains unexplored.

Considering that a proinflammatory ocular surface milieu favors TRPV1 activation and that overstimulation of TRPV1 channels may have deleterious effects on cell membrane integrity and function according to *in vitro* models,^39,40^ the starting hypothesis for this study was that excessive TRPV1 signaling contributes to corneal nerve damage in DED. To this aim, changes in corneal nerve function and morphology were compared between wild-type and *Trpv1*-knockout (*Trpv1*KO) mice with DED. The effect of pharmacologic TRPV1 blockade on the corneal nerves of wild-type mice with DED was also assessed. Altogether, the results herein showed that ocular TRPV1 activity is required for corneal nerves to be affected in DED, which has considerable therapeutic implications.

## Materials and Methods

### Mice

C57BL/6 (C57BL/6NCrl) mice were originally obtained from Charles River Laboratories (Wilmington, MA). *Trpv1*KO (B6.129X1-*Trpv1^tm1Jul^*/J; JAX stock number 003770) and recombination activating gene 1-knockout (*Rag1*KO; B6.129S7-*Rag1^tm1Mom^*/J; JAX stock number 002216) mice were purchased from The Jackson Laboratory (Bar Harbor, ME). Mice were bred and maintained at the Institute of Experimental Medicine’s animal facility. All mice were 6 to 8 weeks old at the beginning of the experiments, and both male and female mice were included. All protocols were approved by the Institute of Experimental Medicine animal ethics committee (approval number 084/2020) and adhered to the Association for Research in Vision and Ophthalmology Statement for the Use of Animals in Ophthalmic and Vision Research.

### Reagents, Antibodies, and Cell Cultures

All chemical and biological reagents were from Sigma-Aldrich (Buenos Aires, Argentina) unless otherwise speci-fied. All antibodies and the most significant reagents are listed in Table 1. All cell cultures were done in RPMI 1640 medium supplemented with 10% fetal calf serum, 10 mmol/L glutamine, 100 U/mL penicillin, 100 μg/mL streptomycin, and 5 × 10^−5^ mol/L 2-mercaptoethanol in a humidified incubator with 5% CO_2_ at 37°C.

### Lacrimal Gland Excision Surgery

Mice were anesthetized by i.p. injection of ketamine (100 mg/kg) and xylazine (10 mg/kg) and placed on a heated pad. Excision surgery comprised four steps: first, a 3-mm–long incision was made along the middle third of the line joining the lateral canthus of the ear and the pinna; second, the superior pole of the extraorbital lacrimal gland was exposed by incising the ensheathing fibrous capsule; third, the lacrimal gland was pulled out gently and excised, taking special care not to damage the blood vessels next to its inferior pole; and fourth, the skin was closed using 6-0 nylon thread. The glands from both sides were excised sequentially. Sham surgery consisted of only steps 1 and 4. In all cases, a single dose of 10 mg/kg diclofenac sodium was injected subcutaneously in the scruff for postoperative analgesia, and ciprofloxacin ointment was applied over the wound once the surgery was completed. The eyes were protected from desiccation with 0.4% sodium hyaluronate (Dropstar LC, Laboratorio Poen, Argentina) until the mice recovered from anesthesia.

### Assessment of Tear Production

Tear production was measured by inserting a 1-mm–wide phenol-red impregnated filter paper strip in the inferior conjunctival fornix adjacent to the lateral canthus, where it was held in place for 60 seconds while restraining the mouse gently and allowing for normal blinking. The wetted length of the right eye of each mouse was measured and used as a data point.

### Assessment of Corneal Epithelial Barrier Function

Corneal fluorescein uptake was measured as previously described.^48^ In brief, 0.5 μL of dextran-fluorescein isothiocyanate [average molecular weight, 3000 to 5000; 10 mg/mL in phosphate-buffered saline (PBS)] was applied to each eye and then the mouse was returned to its cage. After 3 minutes, a 10-second–long video of each eye under blue light was captured with the aid of a fluorescence-adapted dissection microscope (NightSea SFA-RB; Electron Microscopy Sciences, Hatfield, PA). For analysis, a masked observer (M.P. and A.V.) exported a representative video frame as an image and selected the corneal area suitable for analysis, excluding reflections and other artifacts, using ImageJ software version 2.15.0 (NIH, Bethesda, MD; https://imagej.net/software/fiji). Then, the green channel was extracted, the mean fluorescence intensity within the resulting region of interest was calculated after background subtraction (50-pixel rolling ball radius), and the average of both eyes was used for analysis.

### Assessment of Corneal Mechanical Sensitivity

Mechanical thresholds were determined using a mouse-adapted version of Cochet-Bonnet esthesiometry.^7,34^ Nylon 6-0 monofilament was cut into segments of varying lengths (1.0 to 5.5 cm in 0.5-cm steps). With the mouse held firmly in one hand, the cornea was touched six times with each filament, starting with the longest segment. A positive response was defined as blinking and retraction of the eye in reaction to at least three of the six tries. The longest segment yielding a positive response was used as the sensitivity threshold, and the average of both eyes was used for analysis. Corneal sensitivity was measured in the morning (8 to 11 AM) before any other experimental handling.

### Eye-Closing Ratio

One mouse at a time was placed on an elevated platform and allowed to habituate for 2 minutes. Then, a >1-minute–long video was recorded with a camera placed at the same height. For analysis, a masked observer (M.P.) selected snapshots in which each eye was clearly visible. The distance between canthi (x) and between the upper and lower lids (y) was measured using ImageJ software, and then the corresponding eye-closing ratio was calculated as y/x. At least two snapshots per eye were analyzed, and then the results from both eyes were averaged to obtain one data point per mouse.

### Capsaicin Sensitivity

Eye wiping behavior was measured in response to 100 μmol/L capsaicin. Immediately after applying 5 μL solution onto each eye, the mouse was placed in a separate cage and recorded with a camera placed above for at least 30 seconds. For analysis, a masked observer (M.P.) counted the number of eye wipes during the first 30 seconds using a slow playback speed.

### Collection of Eye Tissue

After euthanasia, the conjunctival tissue of each eye was excised as two strips (superior and inferior) under a dissection microscope and collected in ice-cold RPMI 1640 media without serum. Immediately after, enucleation was performed by gently proptosing the eye globe and cutting the optic nerve with curved scissors. The two eyes of each mouse were collected in ice-cold fixative solution. Mice were euthanized one at a time so that all ocular tissue was collected within 5 minutes of the time of death to ensure adequate corneal nerve preservation.^49^

### Corneal Immunostaining

Eyes were processed as described by Tadvalkar et al.^49^ In brief, eyes were fixed in a prechilled formaldehyde-containing buffer for 75 minutes, washed, and stored in methanol at −20°C until processed for staining. Then, the fixed corneas were cut from the back of the eye under a dissection microscope, permeabilized with a graded methanol–Triton X-100 series, blocked overnight with 1% bovine serum albumin and 1% goat serum in PBS, and stained overnight with fluorochrome-conjugated anti-tubulin β3 and CD324 (E-cadherin) antibodies. Each batch of anti-tubulin β3 antibody was titrated before use to minimize background staining, usually resulting in 0.5 to 0.7 μL antibody/200 μL buffer/cornea (2.5 to 3.5 μg/mL) as optimal. The stained corneas were washed three times for 60 minutes in PBS-Tween 0.02%, counterstained with 1 μg/mL DAPI, mounted flat with the aid of relaxing cuts in Aqua-Poly/Mount (PolySciences, Warrington, PA), and stored at 4°C until imaged.

### Confocal Laser-Scanning Microscopy Acquisition

Image acquisition was performed with a FluoView FV1000 confocal microscope (Olympus, Tokyo, Japan) equipped with Plapon 60 × /1.42 and UPlanSapo 20 × /0.75 objectives. Z stacks (0.5-μm step size) spanning the entire corneal epithelium (approximately 30 μm) were obtained first at the corneal center (defined as the center of the nerve whorl or as the center of the disorganized area in those samples with highly disrupted nerve whorls), and then at two opposite locations 600 μm from the center. Corneal nerve analysis was performed at three different levels within the corneal epithelium. For subapical nerve terminals, the first section located entirely beneath the apical epithelial squames (1 to 1.5 μm deep, usually the third or fourth) was selected. Then, the image was thresholded after background subtraction (10-pixel rolling ball radius), and the percentage area occupied by nerve endings was determined by the corresponding ImageJ software function. For midepithelial nerve terminals, the midsection (× 60) between the apical- and basal-most sections from each stack was chosen. Then, the number of nerve endings was assessed after background sub-traction (10-pixel rolling ball radius) by a masked observer using the Cell Counter ImageJ software function. Data are shown as the number of terminals/× 60 field (423.94 μm^2^ area). To analyze the complexity of the subbasal epithelial nerves, the Sholl plugin in ImageJ software was used. In brief, a maximum intensity projection of the 10 sections encompassing the corneal subbasal nerve mat was generated, then the background was subtracted (50-pixel rolling ball radius), and the image was thresholded. Finally, 10 concentric circles with a 10-μm radius step size were traced at the center of the final image, and the resulting sum of intersections of tubulin β3^+^ nerves for each concentric circle was calculated using the software and used for analysis.^38^ Corneal epithelial nuclei were counted using the built-in neural network model for fluorescent nuclei of the ImageJ software plugin StarDist, a cell/nuclei detection method.^50^ The nuclei count in 30 sections spaced 1 μm apart starting at the basal-most epithelial section and progressing apically were added to obtain one data point.

### Real-Time Quantitative PCR Analysis of Trigeminal Gene Expression

Euthanized mice were transcardially perfused with phosphate-buffered saline. Both trigeminal ganglia from each mouse were isolated under a dissection microscope, collected in ice-cold Trizol, and stored at −70°C as one sample. RNA was extracted with Direct-zol RNA MiniPrep columns (Zymo Research, Irvine, CA) as per the manufacturer’s instructions, and reverse transcription was performed as previously described.^38^ Real-time quantitative PCR was performed with 50 ng cDNA, SsoFast EvaGreen Supermix (Bio-Rad Laboratories, Hercules, CA), and primers in a final reaction volume of 20 μL. Primers (Table 2) were designed using the Primer3 software, purchased from Ruralex-Fagos (Buenos Aires, Argentina), and used at 400 Nm. The reaction was performed in a CFX Connect Real-Time PCR Detection System (Bio-Rad Laboratories). The glyceraldehyde 3-phosphate dehydrogenase gene was used for normalization of the results for each sample and then the fold change in specific mRNA levels between groups was calculated by using the 2^–ΔΔCt^ method.

### Preparation and Flow Cytometry Analysis of Conjunctival Cell Suspensions

Conjunctival tissue was minced into fragments with the aid of scissors, incubated in collagenase (1 mg/mL) in PBS at 37°C with gentle shaking for 30 minutes, then DNAse (2 U/mL) was added and the tissue samples were digested for another 15 minutes. Digestion was stopped by adding 2 mmol/L EDTA and 10% fetal calf serum, and the suspension was washed and filtered for staining. Cell suspensions were first stained with a fixable viability dye, washed, Fc receptor blocked, stained for surface markers CD45, CD4, and Ly6G, and then fixed in 1% paraformaldehyde in PBS. The entire cell suspension resulting from one mouse was stained and acquired for analysis as one independent sample. First, singlets were gated based on forward scatter height versus area, then gated on side scatter height versus side scatter area, then gated on viability dye-excluding events (viable cells), and finally on CD45 ^+^ CD4 ^+^ or CD45 ^+^ Ly6G ^+^ events.

### Spleen and Lymph Node Cells

Cervical lymph nodes alone (for flow cytometry studies) or combined with axillary and inguinal lymph nodes (for adoptive transfer studies) were harvested after euthanasia and rendered into a cell suspension by mechanical dissociation through nylon mesh. Splenocyte suspensions were prepared in the same way, followed by red blood cell lysis with ammonium chloride–potassium buffer, and then pooled with lymph node cells for CD4^+^ T-cell isolation.

### Intracellular Cytokine Staining

Cells were stimulated in U-bottom 96-well plates (0.5 × 10^6^ cells/0.2 mL media/well) for 5 hours with 50 ng/mL phorbol myristate acetate, 1 μg/mL ionomycin, and 10 μg/mL brefeldin A. DNAse (1 U/mL) was added 15 minutes before the stimulation period ended. The cells were then washed, stained with a fixable viability dye, washed, surface stained for CD4, and then fixed, permeabilized, and stained intracellularly for cytokines interferon (IFN)-γ and IL-17A with the Cyto-Fast Fix/Perm Buffer Set (number 426803; BioLegend, San Diego, CA) as per the manufacturer’s instructions.

### Isolation and Adoptive Transfer of CD4^+^ T Cells

CD4^+^ cells were isolated from pooled splenocytes and lymph node cells with the aid of magnetic beads (MojoSort Mouse CD4^+^ T Cell Isolation Kit; BioLegend; number 480033) as per the manufacturer’s instructions. Cell purity was >95% as assessed by flow cytometry (CD4 staining). For adoptive transfer experiments, cells were resuspended in PBS and 1 × 10^6^ cells/0.5 mL were injected intraperitoneally into each *Rag1*KO recipient mouse.

### Statistical Analysis

The *t*-test and one- or two-way analysis of variance with Sidak *post hoc* tests were used to compare the means of two or more samples, respectively. Significance was set at *P* < 0.05, and two-tailed tests were used in all experiments. Calculations were performed using GraphPad Prism soft-ware version 7 (GraphPad Software, La Jolla, CA).

## Results

### TRPV1 Deficiency Does Not Impact Tear Production or Corneal Epithelial Integrity in DED

**Figure 1:**
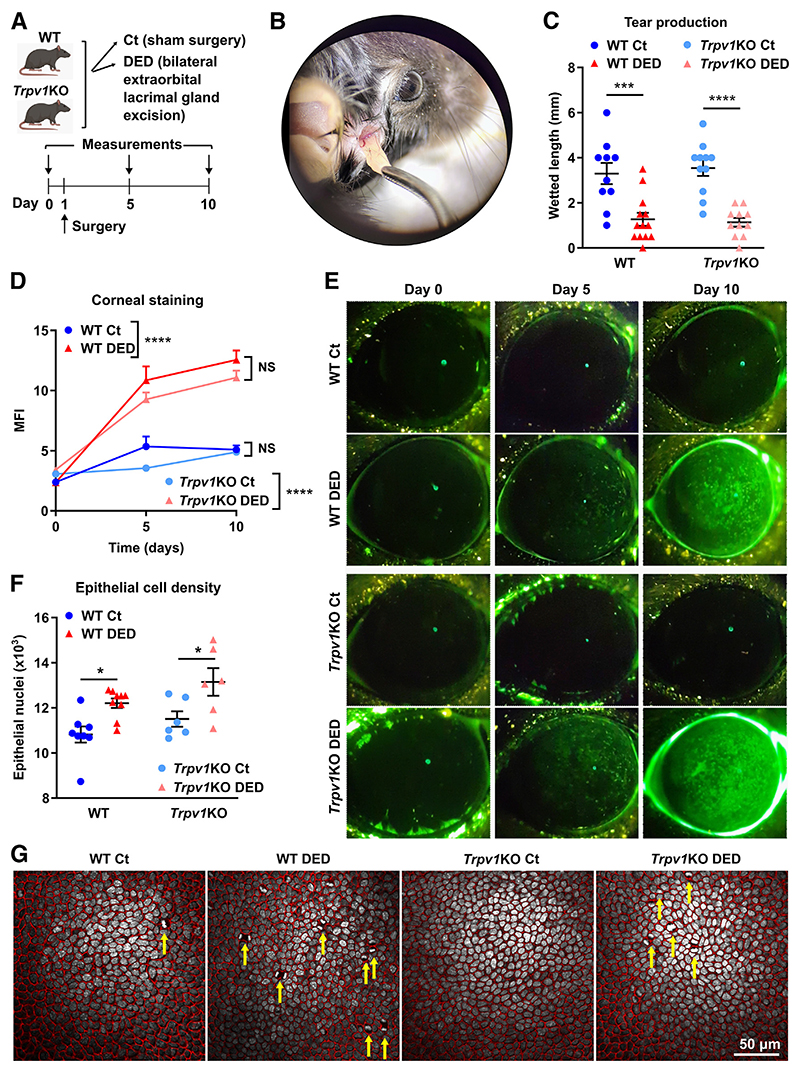
Transient receptor potential vanilloid-1 (TRPV1) deficiency does not impact tear production or corneal epithelial integrity in the context of dry eye. **A:** Dry eye disease (DED) was surgically induced in wild-type (WT) or TRPV1-knockout (*Trpv1*KO) mice through bilateral excision of the extraorbital lacrimal gland. Sham-operated animals were included as controls (Ct). **A:** Experimental design. **B:** Surgical technique. **C:** Tear production on day 5, as measured by phenol red-paper wetting length. **D:** Cumulative data. **E:** Representative micrographs of corneal dextran-fluorescein uptake in Ct and DED mice from both strains. Data shown as the mean fluorescence intensity (MFI), calculated with ImageJ software version 2.15.0 (see *Materials* *and* *Methods*). F: Total nuclei count in 30 sections spanning 30 μm of corneal epithelium. G: Representative micrographs of basal corneal epithelium stained with E-cadherin (red) and DAPI (white). Mitotic figures are highlighted with **yellow arrows**. All experiments were performed twice or more with six mice per group per experiment. **C, D**, and **F:** To compare means, two-way analysis of variance was used for strain and treatment (**C** and **F**) and group and time (**D**). Sidak *post hoc* test was applied in all cases. Data are given as means ± SEM (**C, D**, and **F**). *P < 0.05, ***P < 0.001, and ****P < 0.0001. Scale bar = 50 μm (**G**). NS, not significant.

Among other stimuli, TRPV1 channels are activated by endogenous lipid-derived inflammatory mediators and hyperosmolar conditions,^51^ both of which are found in the DED-affected cornea. In line with this, tear hyper-osmolarity, the natural consequence of increased desiccation in DED, enhances TRPV1 signaling in the cornea.^31^ Also, TRPV1 activation after corneal alkali burn elicits neurogenic inflammation,^38^ and TRPV1 overstimulation may lead to cell membrane disruption *in vitro*.^39,40^ Therefore, the initial hypothesis for this study was that TRPV1 overactivity contributes to DED-associated corneal nerve degeneration. To test this, the development of DED in wild-type and *Trpv1*KO mice (Figure 1A) was compared using a previously characterized surgical model of the disease (Figure 1B) that involves bilateral excision of the extraorbital lacrimal gland.^52–54^ This model corresponds to an aqueous-deficient form of DED because the mice exhibit a steady, marked reduction in basal tear production.

Although basal tearing is regulated by transient receptor potential melastatin 8 channels expressed in corneal cold thermoreceptors,^55^ there is evidence of TRPV1-mediated sensitization of these cooling-responsive fibers in DED.^56^ Therefore, that ocular surface desiccation was comparable in both strains was verified to rule out a confounding effect of TRPV1 deficiency in this model. To this end, tear production levels were assessed on postoperative day 5 (Figure 1C). Tear production in sham-operated mice of both strains did not differ, as reported elsewhere.^44^ Consistently, lacrimal gland excision reduced tear production in wild-type and *Trpv1*KO mice comparably. Thus, the type of mouse strain had no effect on the tear production observed in both treatments. Next, the development of corneal epitheliopathy was evaluated as a hallmark of DED. Corneal epithelial damage can be graded by assessing the uptake of a fluorescent probe (Figure 1D) as an indicator of corneal epithelial barrier function.^57^ As shown in Figure 1E, corneal staining increased comparably in wild-type and *Trpv1*KO mice with DED, and there was no significant difference between strains at any time point. Finally, epithelial morphology was examined in corneal whole mounts because DED increases cell turnover, which is accompanied by a decrease in epithelial cell size.^58,59^ As shown in Figure 1F, both wild-type and *Trpv1*KO mice with DED had more epithelial cells within the same volume of analyzed corneal epithelium. This was corroborated by the presence of more mitotic figures in the basal epithelial layers of mice with DED from both strains (Figure 1G). *Trpv1*KO mice exhibited higher nuclei counts than wild-type animals. Both findings point toward comparably increased cell proliferation in response to desiccation in both strains. Together the data show that DED-associated changes in tear production and in the corneal epithelium were comparable in wild-type and *Trpv1*KO mice.

### TRPV1 Deficiency Prevents DED-Induced Impairment of Corneal Nerve Function

**Figure 2:**
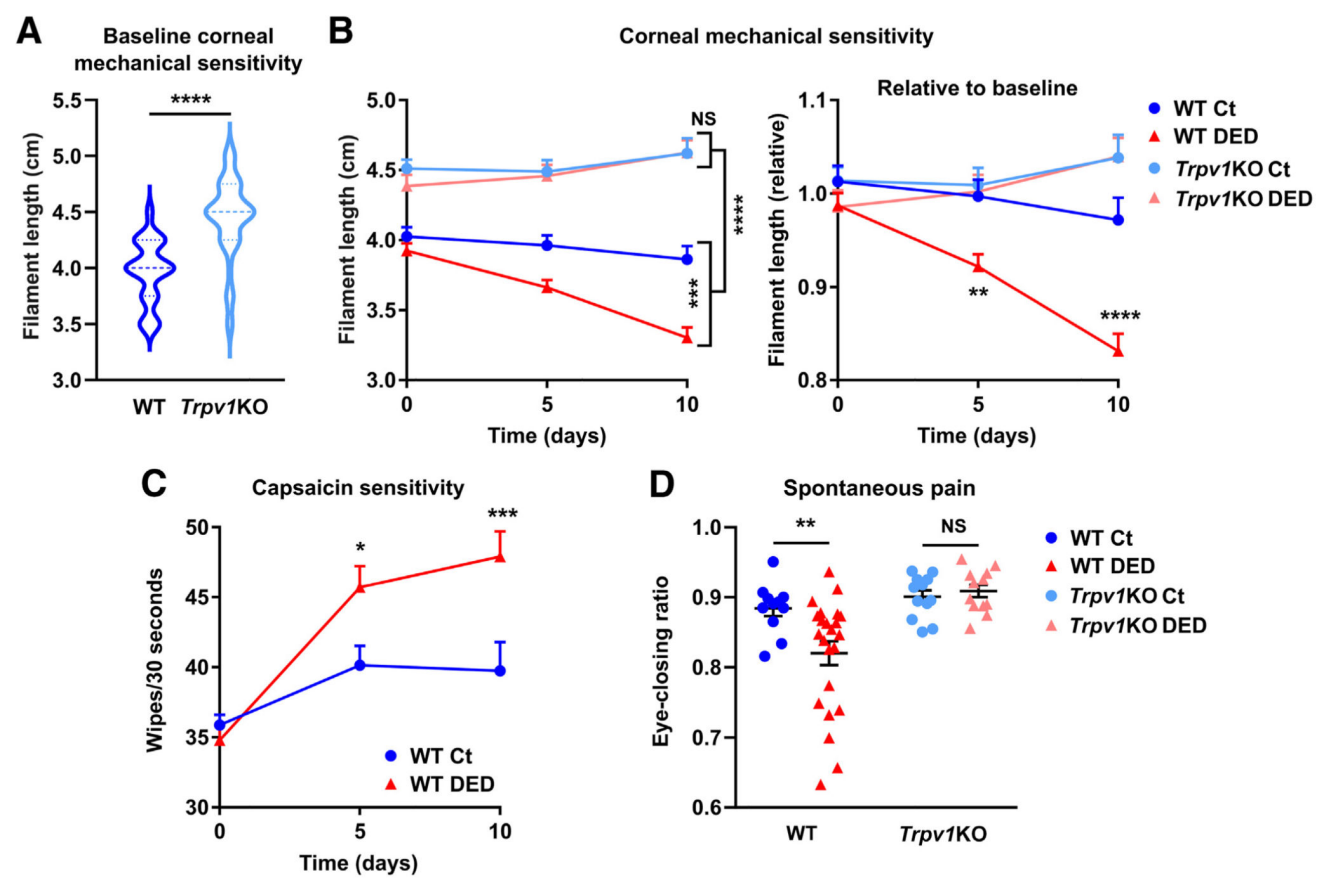
Transient receptor potential vanilloid-1 (TRPV1) deficiency prevents dry eye–induced impairment of corneal nerve function. **A:** Corneal mechanosensitivity was measured in wild-type (WT) or TRPV1-knockout (*Trpv1*KO) mice before each experiment. Pooled data from 43 WT and 47 *Trpv1*KO mice. **B:** Corneal mechanosensitivity in WT and *Trpv1*KO mice with surgically induced dry eye disease (DED) or sham surgery (Ct). Measurements are shown as absolute data (**left panel**) or relative to the same-strain baseline (**right panel**). **C:** Capsaicin sensitivity as measured by the number of eye wipes elicited over 30 seconds following the TRPV1 agonist instillation on both eyes. **D:** Eye-closing ratio measurements in Ct and DED mice from both strains. All experiments were performed twice or more with six mice per group per experiment. **A**–**D:** To compare means, *t*-test was applied (**A**), and two-way analysis of variance with Sidak *post hoc* test was used for group and time (**B** and **C**) and strain and treatment (**D**). Data are given as means ± SEM (**A**–**D**). *P < 0.05, **P < 0.01, ***P < 0.001, and ****P < 0.0001. NS, not significant.

Wild-type and *Trpv1*KO mice with DED developed comparable ocular desiccation and corneal epitheliopathy, thus the extent of DED-induced corneal nerve impairment was compared in both strains. First, corneal mechanical sensitivity was measured using a mouse-adapted version of Cochet-Bonnet esthesiometry, a widely used technique to gauge corneal nerve function in the clinic that relies on the fact that longer filaments exert less pressure on corneal contact (Figure 2A). Unexpectedly, *Trpv1*KO mice had a significantly lower pressure threshold at baseline, indicating greater sensitivity to mechanical stimulation than wild-type mice. As previously reported,^21^ wild-type mice with DED showed a progressive decline in mechanosensitivity on days 5 and 10 compared with wild-type controls (Figure 2A). By contrast, *Trpv1*KO mice with DED did not experience a change in their corneal mechanosensitivity thresholds. This finding indicates that wild-type mice experienced more DED-associated corneal nerve dysfunction than *Trpv1*KO mice. Considering the different starting thresholds between strains, relativization of measurements to the same-strain baseline better showed that the DED-associated change occurred only in wild-type mice (Figure 2B). Corneal sensitivity to capsaicin, a well-characterized agonist of TRPV1 channels that elicits ocular pain and nocifensive behavior in mice (Supplemental Video S1), was also measured to ascertain the function of other corneal nerve endings. Over a 10-day period, capsaicin instillation elicited a constant number of wipes in sham-operated wild-type mice, whereas this nocifensive response progressively increased in wild-type mice with DED (Figure 2C). This finding indicates that DED brings about sensitization of TRPV1-expressing corneal fibers, suggesting that TRPV1 signaling increases with inflammation during disease progression. As expected, capsaicin did not evoke a wiping response in *Trpv1*KO mice (Supplemental Video S2). Finally, the eye-closing ratio (Figure 2D) was analyzed as part of the validated Mouse Grimace Scale for quantifying the spontaneous subjective pain experience in mice.^60,61^ Wild-type mice with DED showed signs of spontaneous pain, whereas *Trpv1*KO mice with DED did not exhibit differences in the eye-closing ratio compared with sham-operated mice of both strains. This result indicates that DED brings about TRPV1-dependent nociception that ultimately results in pain sensation.

Altogether, these findings show that DED causes TRPV1 sensitization and activation, and more importantly, that these events are accompanied by corneal nerve dysfunction.

### TRPV1 Deficiency Reduces DED-Induced Changes in Corneal Nerve Structure

**Figure 3:**
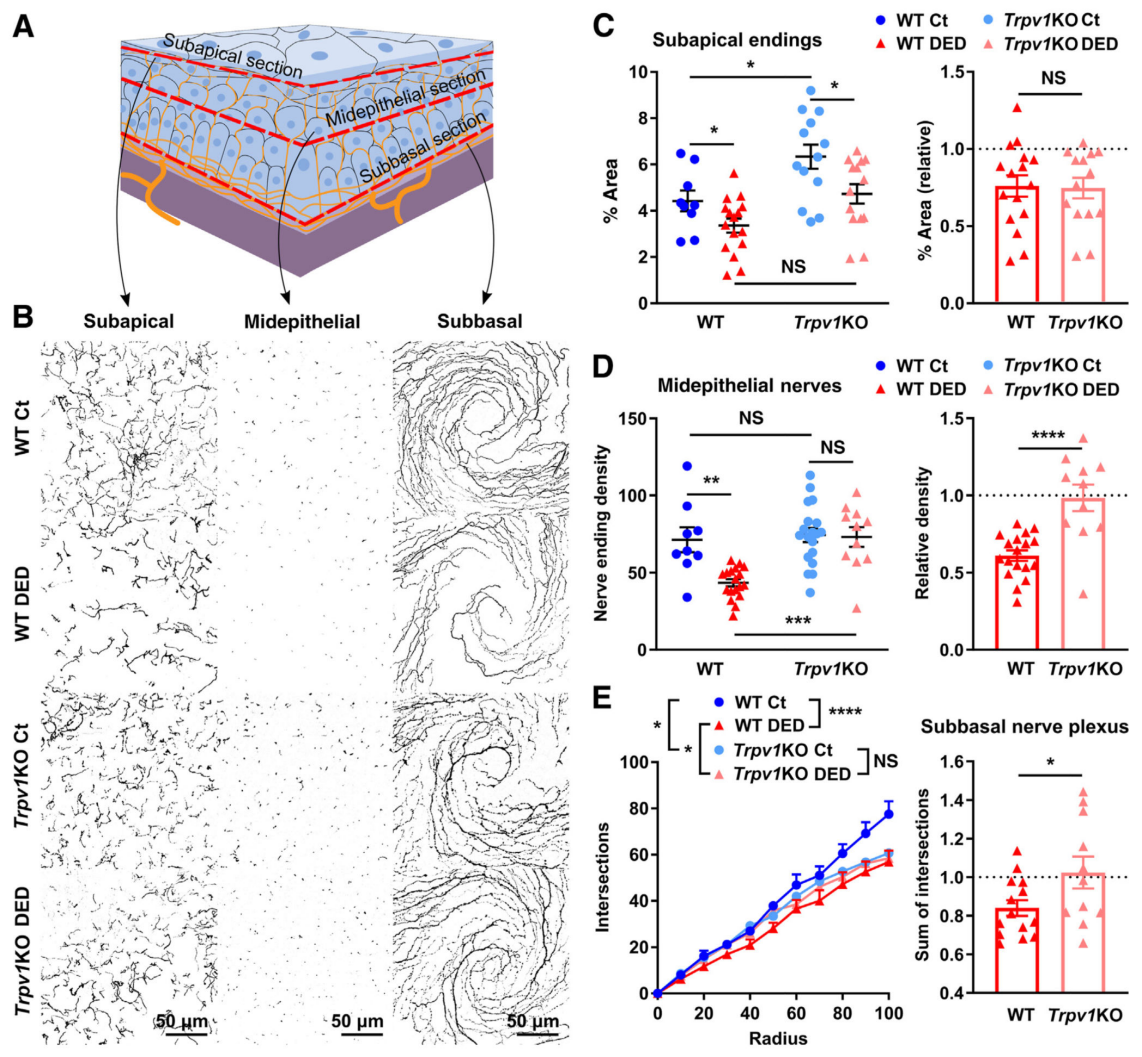
Transient receptor potential vanilloid-1 (TRPV1) deficiency reduces dry eye disease (DED)–induced changes in corneal nerve structure. Corneal whole mounts from wild-type (WT) or TRPV1-knockout (*Trpv1*KO) mice with surgically induced DED or sham surgery (Ct) were stained with Alexa Fluor 488 antitubulin β3 (green or white). **A:** Schematic of the levels (subapical, midepithelial, and subbasal) at which corneal nerve morphology was analyzed. **B:** Representative micrographs for each level from all groups. **C:** Quantification (percentage area) of the most superficial nerve terminals imaged *en face* beneath the apical epithelial squames (subapical section). **D:** Density (count of nerve endings/field) of intraepithelial corneal nerves in cross-section at the midepithelial level as they run perpendicularly to the surface. **C** and **D:** Measurements are shown as absolute data from all groups (**left panels**) or the DED group from each strain relative to the same-strain Ct group (**dashed lines** indicate Ct group values; **right panels**). **E:** Quantification of corneal neural complexity at the subbasal level by Sholl analysis. Absolute number of intersections at each Sholl radius from all groups (**left panel**) or sum of intersections of the DED groups from both strains relative to the same-strain Ct group (**dashed line** indicates Ct group values; **right panel**). All experiments were performed twice or more with six mice per group per experiment. **C**–**E:** To compare means, two-way analysis of variance with Sidak *post hoc* test was used in the **left panels** for treatment and strain (**C** and **D**) and group and radius (**E**), whereas the one-sample *t*-test was applied in the **right panels**. Data are given as means ± SEM (**C**–**E**). *P < 0.05, **P < 0.01, ***P < 0.001, and ****P < 0.0001. Scale bars = 50 μm (**B**). NS, not significant.

As no DED-associated corneal nerve dysfunction was observed in *Trpv1*KO mice, nerve morphology was examined by confocal microscopy of corneal whole mounts. The architecture of the intraepithelial corneal nerves was qualitatively comparable between sham-treated wild-type and TRPV1 Channels in Corneal Nerve Damage *Trpv1*KO mice, with the typical whorl pattern of the subbasal plexus. Axial reconstructions of the central intraepithelial corneal nerves (Supplemental Figure S1) confirmed the conserved morphology in sham-treated *Trpv1*KO mice. Compared with sham-treated mice, reconstructions from wild-type mice with DED showed focal reductions of innervation that were more evident at the midepithelial level, where the nerve fibers run perpendicularly toward the corneal surface (Figure 3A). *En face* imaging at three distinct depths (Figure 3B and Supplemental Figure S2) within the corneal epithelial layer allowed for quantification and comparison between groups (Figure 3, C–E). Baseline innervation at some levels differed between strains: *Trpv1*KO mice have higher subapical nerve-occupied area, comparable midepithelial nerve density, and lower subbasal nerve complexity. To account for this source of variation and thereby to better reflect DED-induced changes, data were relativized to sham-treated mice of the same strain. As shown in Figure 3C, DED comparably decreased the total area occupied by subapical nerve endings beneath the apical-most layer of corneal epithelial cells in wild-type and *Trpv1*KO mice. By contrast, wild-type mice with DED had a reduction in the midepithelial and subbasal nerve density, whereas *Trpv1*KO mice with DED did not (Figure 3, D and E). Altogether, these results show that the pattern and extent of DED-associated morphologic nerve changes in *Trpv1*KO mice is not the same as in wild-type mice. Despite baseline differences, DED induces a comparable loss of the most exposed subapical endings in both strains, whereas the deeper intraepithelial nerves remain relatively unchanged in *Trpv1*KO mice.

### TRPV1 Deficiency Reduces DED-Induced Changes in Corneal Nerve Structure

**Figure 4:**
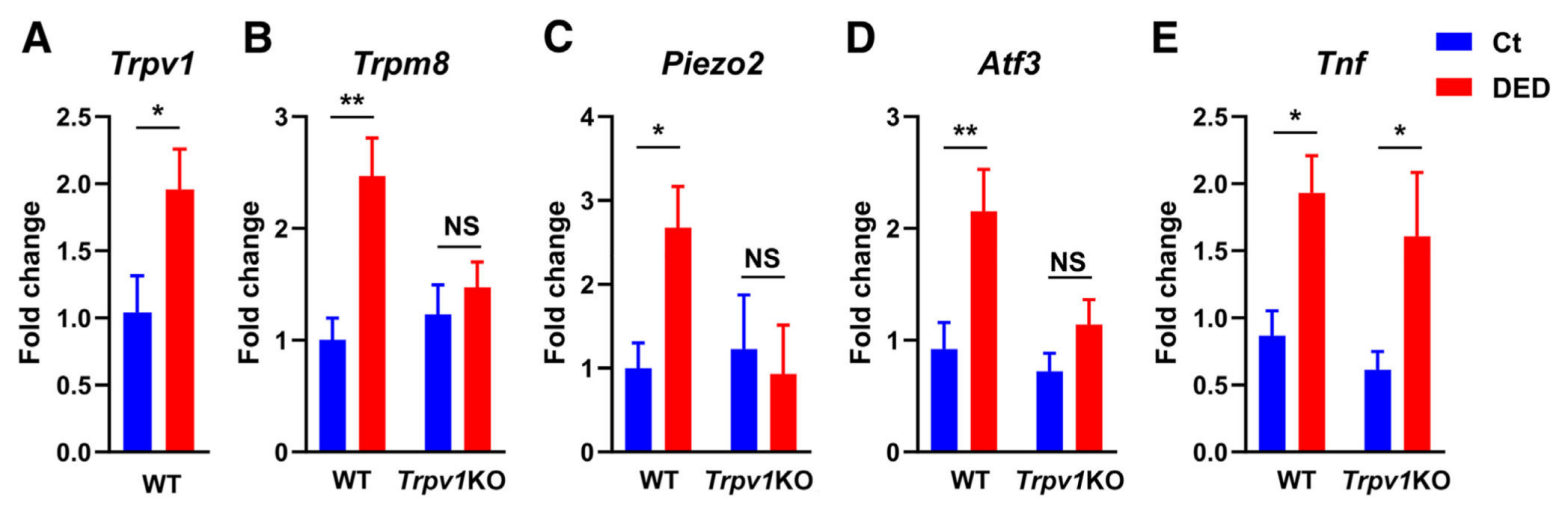
Transient receptor potential vanilloid-1 (TRPV1) deficiency attenuates dry eye disease (DED)–induced gene expression changes in the trigeminal ganglion. Trigeminal ganglia from wild-type (WT) or TRPV1-knockout (*Trpv1*KO) mice with surgically induced DED or sham surgery (Ct) were harvested on day 10 and assayed for mRNA expression levels of *Trpv1* (**A**), transient receptor potential melastatin-8 (*Trpm8*; **B**), *Piezo2* (**C**), activating transcription factor-3 (*Atf3*; **D**), and tumor necrosis factor (*Tnf*; **E**) genes. **A**–**E**: To compare means, *t*-test was applied (**A**), and two-way analysis of variance with Sidak *post hoc* test was used for treatment and strain (**B**–**E**). Data are given as means ± SEM (**A**–**E**). *n* = 6 or more per group (**A**–**E**). *P < 0.05, **P < 0.01. NS, not significant.

Axonal degeneration in DED induces reactive changes in gene expression in the trigeminal ganglion, where the cell bodies of the cornea-innervating neurons are located.^12,24,47^ As reported by others,^44,45^ a significant increase in *Trpv1* expression was observed in the trigeminal ganglia of wild-type mice after 10 days of DED (Figure 4A). As expected, *Trpv1* expression was not detectable in *Trpv1*KO mice (data not shown). The expression levels of *Trpm8* and *Piezo2*, the two sensory channels typically associated with corneal cold-thermoreceptor and selective mechanonociceptor fibers, were increased in wild-type DED mice (Figure 4, B and C) but not in *Trpv1*KO DED mice (Figure 4, B and C). Consistently, expression levels of activating transcription factor 3 (*Atf3*), a transcription factor that is rapidly up-regulated in sensory neurons in response to axonal injury,^62^ increased in wild-type DED mice (Figure 4D) but not in *Trpv1*KO DED mice. However, expression of tumor necrosis factor (Figure 4E) increased in both wild-type and *Trpv1*KO mice with DED, suggestive of neuroinflammatory changes in response to ocular surface disease. Altogether, these findings show that some reactive changes in trigeminal gene expression observed in wild-type mice with DED are attenuated or absent in *Trpv1*KO mice with DED, which is consistent with the corneal findings. This suggests that TRPV1 expression is involved in corneal nerve impairment.

### Pharmacologic TRPV1 Blockade in Wild-Type Mice with DED Prevents Corneal Nerve Impairment

**Figure 5:**
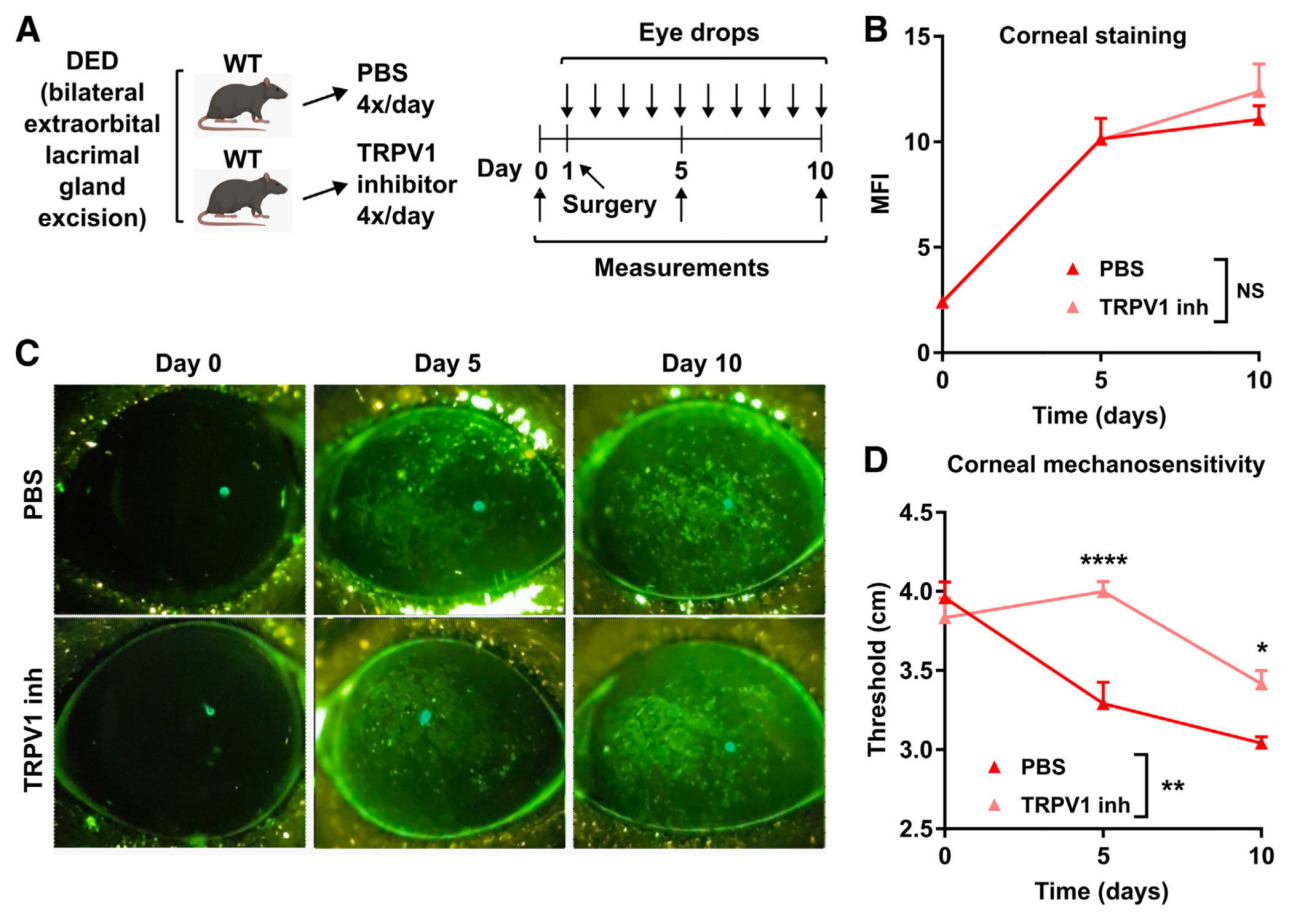
Pharmacologic transient receptor potential vanilloid-1 (TRPV1) blockade in wild-type (WT) mice prevents dry eye disease (DED)–induced decrease in corneal mechanosensitivity. **A:** DED was surgically induced in WT mice receiving eye drops (4 times/day) containing either phosphate-buffered saline (PBS) or 100 μg/mL SB-366791, a known TRPV1 inhibitor (TRPV1 inh). **B** and **C:** Cumulative data (**B**) and representative micrographs (**C**) of corneal dextran-fluorescein uptake in PBS and TRPV1 inhibitor–treated DED mice. **D:** Corneal mechanosensitivity in PBS and TRPV1 inhibitor–treated DED mice. All experiments were performed twice or more with six mice per group per experiment. **B** and **D:** Two-way analysis of variance with Sidak *post hoc* test was used for treatment and time to compare means. Data are given as means ± SEM (**B** and **D**). *P < 0.05, **P < 0.01, and ****P < 0.0001. MFI, mean fluorescence intensity; NS, not significant.

**Figure 6:**
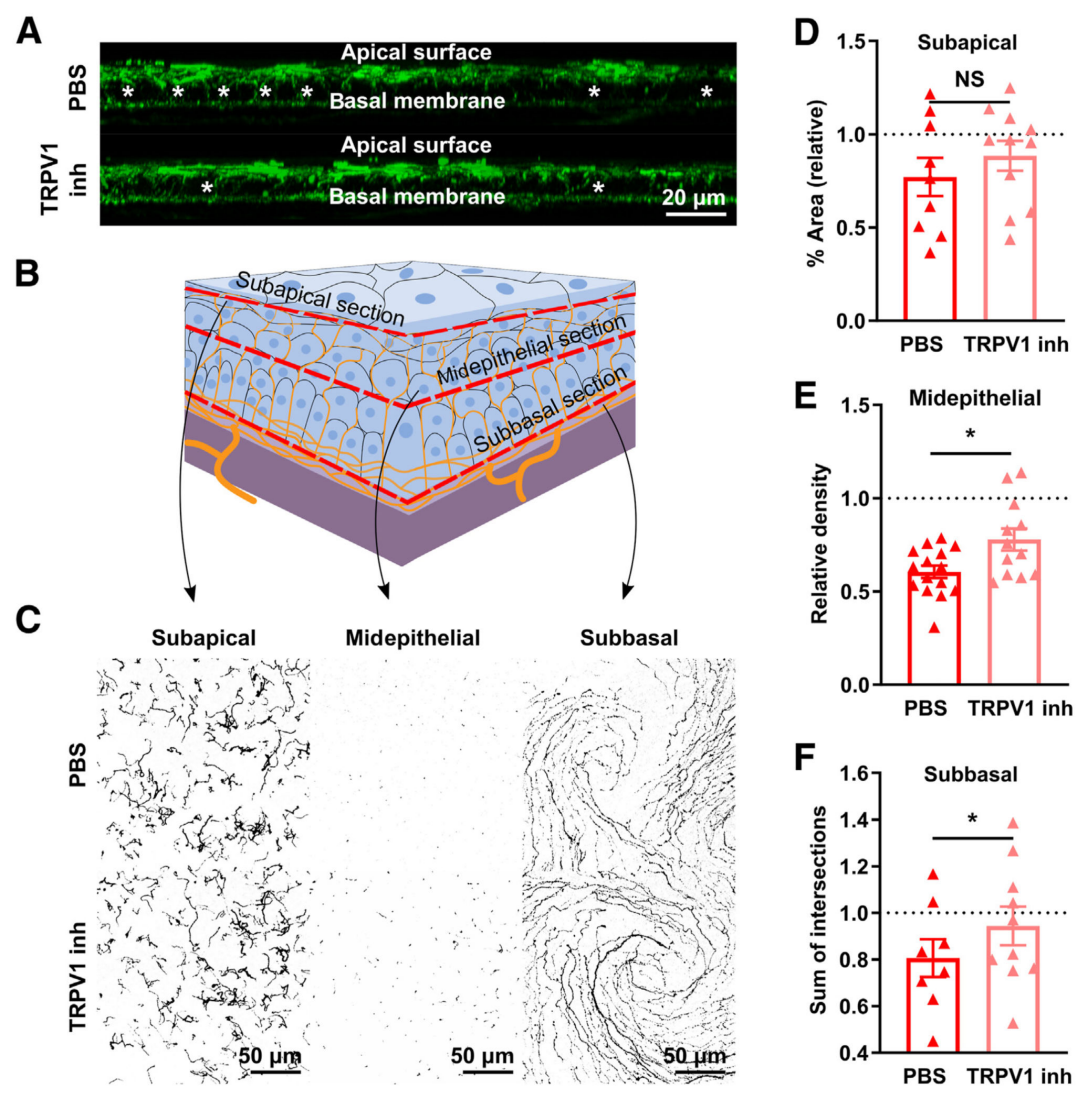
Pharmacologic transient receptor potential vanilloid-1 (TRPV1) blockade in wild-type (WT) mice with dry eye disease (DED) prevents corneal nerve impairment. DED was surgically induced in WT mice receiving eye drops (4 times/day) containing either phosphate-buffered saline (PBS) or 100 μg/mL SB-366791, a known TRPV1 inhibitor (TRPV1 inh). **A:** Axial reconstructions of the central corneal nerves spanning the entire epithelial layer and the anterior stroma (tubulin β3 staining). Representative images from PBS and TRPV1 inh mice. **Asterisks** indicate areas of evident corneal nerve loss. **B:** Schematic of the levels (subapical, midepithelial, and subbasal) at which corneal nerve morphology was analyzed. **C:** Representative micrographs of corneal nerves from both groups at each of the three analyzed levels. **D:** Quantification (percentage area) of the most superficial nerve terminals imaged *en face* beneath the apical epithelial squames (subapical section). **E:** Density (count of nerve endings/field) of intraepithelial corneal nerves in cross-section at the midepithelial level as they run perpendicularly to the surface. **F:** Quantification (sum of intersections) of corneal neural complexity at the subbasal level by Sholl analysis. **D**–**F:** Data are shown relative to sham-treated WT mice (**dashed lines**). All experiments were performed twice or more with six mice per group per experiment. The *t*-test was applied in **D**–**F** to compare means. Data are given as means ± SEM (**D**–**F**). *P < 0.05. Scale bars: 20 μm (**A**); 50 μm (**C**). NS, not significant.

Because *Trpv1*KO mice exhibited baseline differences in corneal nerve function (Figure 2A) and morphology (Figure 3, C–E) that could confound the results, the effect of pharmacologic TRPV1 inhibition was tested in wild-type mice with DED (Figure 5A). To this end, the TRPV1 antagonist SB-366791 was instilled in both eyes of mice following a previously validated dosing scheme (4 times/day; 100 μg/mL).^31,38^ The start of TRPV1 blockade coincided with the surgery and lasted until euthanasia. Corneal epitheliopathy and nerve impairment were quantified as before. In agreement with the findings in *Trpv1*KO mice, ocular TRPV1 blockade had no discernible effect (*P* = 0.56) on the development of DED-induced corneal epitheliopathy in wild-type mice (Figure 5, B and C). By contrast, topical TRPV1 inhibition reduced the corneal mechanical sensitivity decrease associated with DED progression (Figure 5D). Although this finding is in line with the knockout-derived observations (Figure 3), TRPV1 inhibitor-treated mice did experience some loss in corneal mechanosensitivity after 10 days of DED (−11% from baseline), which was still significantly lower than that of saline-treated mice at the same time point (−23% from baseline). As shown in Figure 6A, corneal nerve morphology changes in PBS-treated mice were similar to those previously observed in wild-type mice with DED, whereas TRPV1 inhibitor-treated mice showed better preservation of the midepithelial and subbasal nerve segments, as did *Trpv1*KO mice with DED (Supplemental Figure S1). Representative micrographs obtained at the three different levels within the corneal epithelium better depicted the corneal nerve changes (Figure 6, B and C). Quantification of corneal nerves indicated that TRPV1 antagonist instillation did not cause statistically significant change in the subapical (most superficial) nerve endings (Figure 6D). By contrast, TRPV1 inhibition ameliorated DED-induced nerve fiber loss in the midepithelial vertical fibers (Figure 6E) and in the deeper subbasal nerve plexus (Figure 6F). These results confirmed the findings in *Trpv1*KO mice: ocular TRPV1 activity is associated with corneal nerve impairment but not corneal epitheliopathy development in DED.

### TRPV1 Expression in the Cornea but Not in T Cells Is Relevant for Preventing Corneal Nerve Impairment in DED

**Figure 7:**
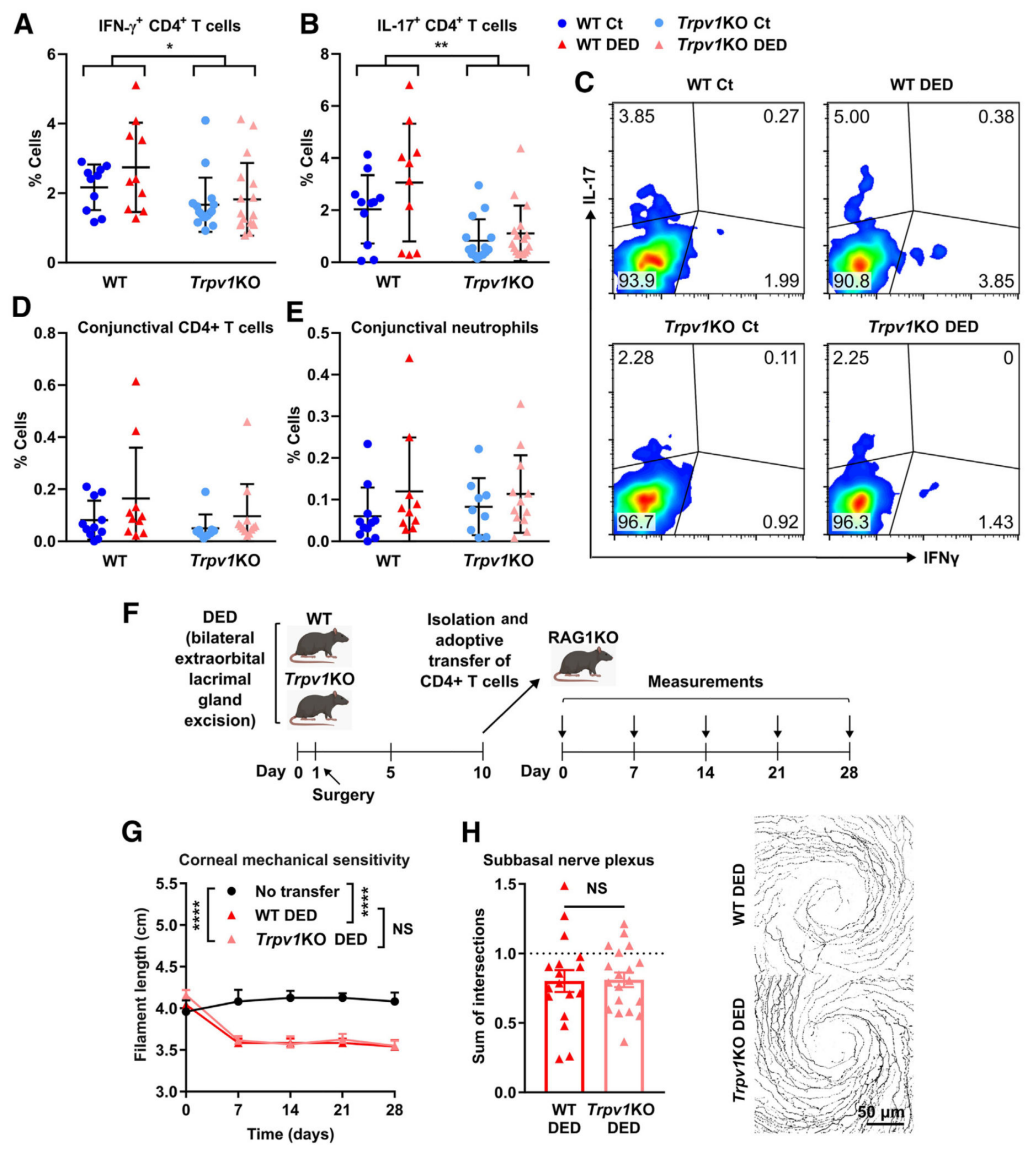
Transient receptor potential vanilloid-1 (TRPV1) signaling in the corneal tissue but not in T cells is relevant for preventing corneal nerve impairment in dry eye disease (DED). DED was surgically induced in wild-type (WT) or TRPV1-knockout (*Trpv1*KO) mice through bilateral excision of the extraorbital lacrimal gland. Sham-operated animals were included as controls (Ct). **A** and **B:** Interferon (IFN)-γ (**A**) and IL-17 (**B**) production, as assessed by flow cytometry of CD4^+^ T cells obtained from the cervical lymph nodes of Ct and DED mice from both strains. **C:** Representative dot plots (IFN-γ versus IL-17) of CD4^+^ T cells from all groups. **D** and **E:** Proportion of CD4^+^ T cells (**D**) and neutrophils (Ly6G^+^; **E**) in conjunctival cell suspensions from all groups, as determined by flow cytometry. **F:** Experimental design of adoptive transfer. DED was surgically induced in WT and *Trpv1*KO mice. After 10 days, CD4^+^ T cells were isolated from spleens and lymph nodes, and 1 × 10^6^ cells/0.5 mL were injected intraperitoneally into each *Rag1*KO recipient mouse. **G:** Corneal mechanosensitivity as measured weekly in adoptively transferred mice. **H:** Quantification (sum of intersections) of neural complexity at the subbasal level by Sholl analysis in corneas obtained 4 weeks after adoptive transfer. Data are shown relative to sham-treated WT mice (**dashed line** indicates Ct group values; **left panel**) along with representative micrographs (**right panels**). All experiments were performed twice or more with six mice per group per experiment. **A, B, D, E, G**, and **H**: To compare means, two-way analysis of variance with Sidak *post hoc* test was used for treatment and strain (**A, B, D**, and **E**) and treatment and time (**G**), whereas *t*-test was applied to compare means in **H**. Data are given as means ± SEM (**A, B, D, E, G**, and **H**). *P < 0.05, **P < 0.01, and ****P < 0.0001. Scale bar = 50 μm (**H**). NS, not significant.

Type 1 helper T cell (Th1) and type 17 helper T cell (Th17) CD4^+^ T cells are pathogenic in DED as they promote apoptosis of corneal epithelial and conjunctival goblet cells and favor corneal barrier disruption.^28–31,63–67^ On the other hand, TRPV1 channels play a role in the activation of CD4^+^ T cells by enabling T-cell receptor–induced calcium influx.^68^ Therefore, Th1 and Th17 CD4^+^ T cells were investigated to rule out a confounding effect of TRPV1 deficiency on their pathogenic capacity. First, IFN-γ and IL-17 production in the CD4^+^ T cells of the eye-draining lymph nodes was measured by flow cytometry (Figure 7, A–C). Wild-type and *Trpv1*KO mice with DED showed a nonsignificant trend toward more IFN-γ– and IL-17−producing CD4^+^ T cells than their same-strain sham-operated littermates. This finding is in line with the modest increase in Th17 CD4^+^ T cells reported in mice, as the change in the pathogenic T-cell response is more qualitative than quantitative,^28,65^ and other non-CD4^+^ T cells are also sources of pathogenic IFN-γ and IL-17 in DED. By contrast, the number of IFN-γ− and IL-17−secreting CD4^+^ T cells was significantly reduced in both groups of *Trpv1*KO mice compared with the wild-type strain. This finding is in line with the previously indicated role of TRPV1 in potentiating CD4^+^ T-cell activation.^68^ As bloodborne immune cells must gain access to the ocular surface through limbal and conjunctival vessels, conjunctival CD4^+^ T cells and neutrophils were quantified by flow cytometry to determine whether TRPV1 deficiency affected conjunctival immune cell recruitment. As shown in Figure 7, D and E, there was no statistically significant difference between groups regarding the extent of CD4^+^and neutrophil infiltration in the conjunctiva, but only a trend toward more cells in DED mice. Altogether these results indicate that Th1 and Th17 CD4^+^ T-cell responses are reduced in *Trpv1*KO mice but that this difference does not appear to be related to DED, as the number of conjunctival CD4^+^ T cells and neutrophils did not differ between strains.

**Figure 8:**
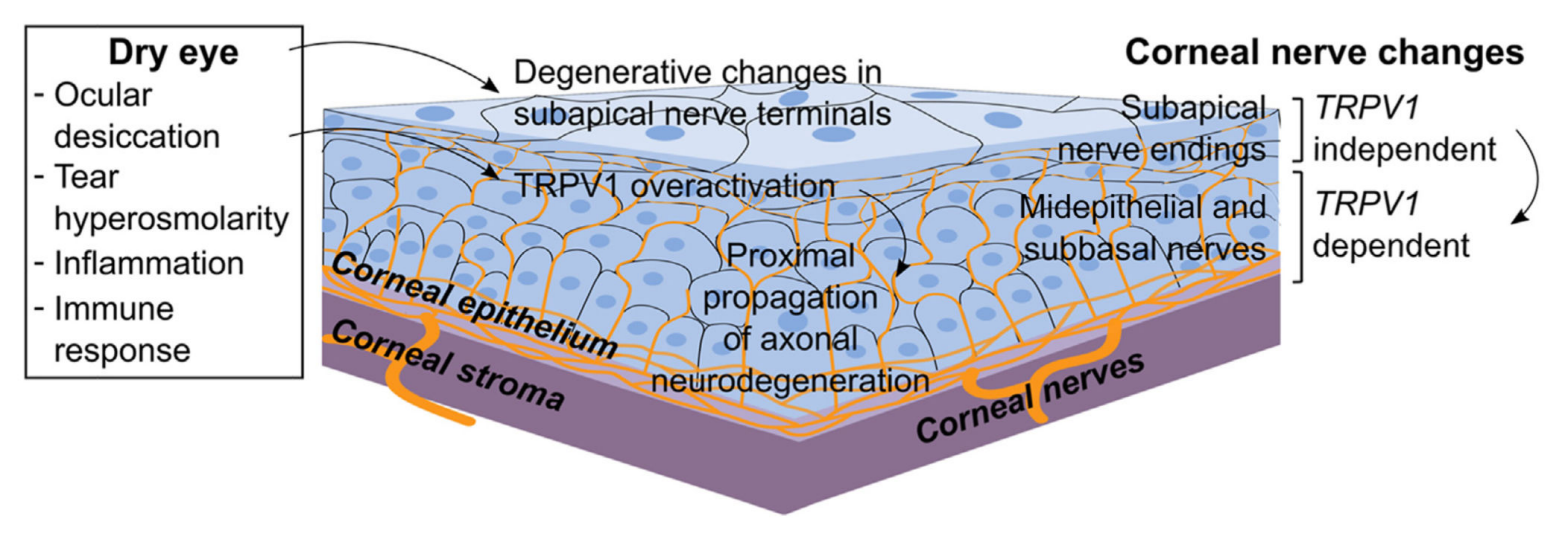
Proposed model for the role of transient receptor potential vanilloid-1 (TRPV1) channels in dry eye disease (DED)–associated corneal nerve impairment. Our findings show that the most superficial corneal nerve endings (subapical nerve terminals) are affected by one or more of the pathogenic factors in DED (ocular desiccation, tear hyperosmolarity, or the inflammatory immune response) independently of the expression/activity of TRPV1 channels. However, extension of corneal nerve changes to the proximal sections (midepithelial and subbasal fibers) of the intraepithelial corneal nerves depends on corneal TRPV1 expression/activity. Thus, corneal TRPV1 channels propagate axonal neurodegeneration in DED.

Because ocular activation of Th1 CD4^+^ T cells independently favors corneal nerve damage,^69^ the protection from DED-induced corneal nerve changes observed in *Trpv1*KO mice could be due to reduced neuropathogenic activity of CD4^+^ T cells in the ocular surface. To rule this out, CD4^+^ T cells from either wild-type or *Trpv1*KO mice with DED were adoptively transferred into T-cell–deficient mice, and their neuropathogenic capacity was compared using the progression of corneal nerve impairment as readout (Figure 7F). As shown in Figure 7G, T-cell–deficient mice reconstituted with CD4^+^ T cells from *Trpv1*KO mice with DED lost corneal mechanical sensitivity to the same extent as wild-type DED CD4^+^ T-cell recipients. Consistent with the commensurable impairment of corneal mechanosensitivity, both groups of recipient mice exhibited similar changes in corneal nerve morphology. As shown in Figure 7H, the complexity of the subbasal nerve plexus (measured by the sum of intersections in Sholl analysis) decreased comparably between wild-type and *Trpv1*KO CD4^+^ T-cell–recipient groups. Altogether, these results show that the lack of TRPV1 expression in CD4^+^ T cells is not responsible for the corneal nerve phenotype summarized in Figures 2 and 3. Therefore, TRPV1 expression within the corneal tissue is involved in the progression of corneal nerve impairment in DED (Figure 8).

## Discussion

DED progression negatively impacts corneal nerves by an as-of-yet incompletely understood mechanism. Here, TRPV1 activity is shown to be required for corneal neurodegeneration to occur in a murine model of DED. TRPV1 deficiency prevents the development of corneal nerve damage, whereas it does not significantly affect the course of DED-induced corneal epitheliopathy. The protective effect of TRPV1 deficiency does not involve an impaired ocular surface immune response nor does it stem from potentially reduced pathogenic activity of *Trpv1*KO CD4^+^ T cells, as their adoptive transfer to T-cell–deficient mice leads to comparable disease as that caused by CD4^+^ T cells from wild-type DED mice. On the contrary, the data point toward a pathogenic role of ocular TRPV1 expression in corneal nerve changes: first, sensitization to capsaicin and TRPV1-dependent pain sensation were observed as DED progresses in the model; and second, a comparable phenotype was obtained by ocular instillation of a TRPV1 blocker. Thus, the findings suggest that TRPV1 channel overactivation in corneal nerves promotes DED-associated corneal nerve impairment (Figure 8).

DED onset is caused by tear film instability, which brings about tear hyperosmolarity and corneal desiccation.^8,9^ The resulting inflammatory ocular surface setting in DED abounds in stimuli that can trigger TRPV1 signaling. TRPV1 channels allow the influx of calcium to the cell on activation by capsaicin, heat, acidic conditions, and endogenous ligands, collectively known as endovainilloids.^70^ Most endovainilloids with TRPV1 bioactivity are cell membrane–derived lipids that are produced during inflammation.^70^ Because of its polymodal nature, TRPV1 plays a key role as an integrator of inflammatory signals in nociception (ie, the detection of damage or potentially threatening stimuli by the nervous system).^71,72^ TRPV1 activity is also regulated indirectly by factors that may increase (sensitize) or decrease (desensitize) the channel’s gating response to agonists.^70,73^ For instance, extracellular ATP released by damaged cells activates surface purinergic receptor P2Y1, triggering a cascade of intracellular events that result in TRPV1 channel phosphorylation and its subsequent sensitization.^74^ Several positive and negative endogenous modulators of TRPV1 are known to be dysregulated in DED.^73,75^ Here, increasing sensitization to capsaicin stimulation was observed in wild-type mice with DED, which suggests that as ocular surface inflammation worsened, the threshold for TRPV1 activation was lowered, and therefore, that TRPV1 signaling increased. Accordingly, TRPV1-dependent spontaneous pain sensation was also recorded in DED mice. This assumption is supported by reports of enhanced corneal TRPV1 activity in other models of DED.^31,44–47^

In contrast to the ample evidence of corneal TRPV1 overactivation taking place in DED, its consequences have not been explored in detail. Corneal TRPV1 expression is demonstrated here to be required for corneal nerve impairment to appear in the context of DED, yet the underlying mechanism remains unknown. As a first possibility, corneal TRPV1 triggering leads to the release of substance P,^38,44^ a proinflammatory neuropeptide that favors dendritic cell maturation and Th1 cell differentiation.^76^ Substance P also modulates IFN-γ production by innate immune cells^77^ and may exert other pathogenic effects on corneal nerves.^78,79^ In line with this, type 1 immunity in the ocular surface is sufficient to drive corneal nerve changes,^69^ and in this study, *Trpv1*KO mice with DED exhibited a trend toward decreased Th1 and Th17 immune responses (Figure 5, A and B). Nonetheless, this finding does not explain the resistance to DED-induced corneal nerve damage in *Trpv1*KO mice because the adoptive transfer of their CD4^+^ T cells promotes corneal nerve impairment in the recipient mice. Therefore, the protective effect must lie in corneal TRPV1 expression. As a second possibility, strong TRPV1 activation triggers the apoptosis of rat cortical neurons *in vitro* through a mechanism that involves extracellular calcium influx, extracellular signal-regulated kinase activation, and reactive oxygen species production.^39^ TRPV1 overstimulation has been shown to induce apoptosis in tumor cells by a similar process that involves mitochondrial instability and caspase activation.^40–43^ However, others have examined TRPV1-expressing neurons in the trigeminal ganglion of mice and rats with DED and found no decrease in their number.^44,45^ Thus, corneal nerve reduction in DED is not likely to be caused by a loss of trigeminal ganglion-residing neurons that supply the eye. A third scenario relates to the interdependency of corneal epithelial cells and intraepithelial nerves.^7,80^ Corneal nerve endings are shed along with their ensheathing epithelial cells following a diurnal cycle,^81^ and on the other hand, neuronal TRPV1 activation inhibits the extension and motility of sensory neurites by regulating microtubule disassembly.^82^ Thus, TRPV1 overactivation might lead to corneal nerve impairment in DED by interfering with the axonal growth required to replenish intraepithelial corneal nerve endings daily. Corneal epithelial cell turnover increases in DED,^58,59^ as confirmed in this model (Figure 1, F and G). Finally, a fourth alternative implicates TRPV1-induced axonal degeneration. Capsaicin application in peripheral tissues causes local ablation of TRPV1-expressing nerve terminals without inducing neuronal death in the sensory ganglia.^83–85^ This pathway is different from capsaicin-induced neuronal apoptosis and involves the activation of calcium-dependent proteases and microtubule depolymerization in the ablated axons.

Multilevel analysis of corneal nerve morphology sheds more light on the pathophysiology of DED-associated corneal nerve changes. In addition to comparable DED-induced corneal epitheliopathy, wild-type and *Trpv1*KO mice evidenced a similar extent of damage in the most superficial nerve endings. These nerve terminals are found interspersed beneath the apical-most layer of corneal epithelial cells, hence the name subapical. The combined effects of ocular desiccation and the ensuing immune response most likely drive these changes that seem to be TRPV1 independent. However, the data also show that the proximal segments of the corneal nerves, which are located deeper within the corneal epithelium, were spared in *Trpv1*KO mice but not in wild-type mice. Thus, although the most superficial (subapical) nerve endings are likely damaged by ocular desiccation and the immune response, TRPV1 overactivation in the same corneal nerve fibers must be involved in the proximal propagation of neurodegenerative changes by any of the mechanisms mentioned in the previous paragraph. Topical blockade of TRPV1 channels afforded more protection to the proximal than to the distal intraepithelial corneal nerve segments in wild-type mice, confirming the distal-to-proximal progression of neural damage in DED. These findings are in line with the more extensive reduction in the apical (distal) than in the basal (proximal) intraepithelial segments of corneal nerves reported by Stepp et al^21^ using a different murine DED model. Whether the TRPV1-activating stimuli are inflammatory or tissue-damage derived remains unknown. Alternatively, TRPV1 channels are responsive to mechanical stimulation, and ocular desiccation increases blinking-associated attrition of the corneal surface.^86^ Therefore, it is possible that deficient corneal lubrication directly contributes to TRPV1 activation in DED. More research is warranted to delineate the underlying pathogenic mechanism of TRPV1-dependent corneal nerve damage reported in this study.

Intriguingly, current data show that *Trpv1*KO mice have higher corneal mechanosensitivity than wild-type mice. By contrast, others have reported that ablation of TRPV1-expressing neurons by different strategies does not affect skin mechanosensitivity.^87–89^ *Trpv1*KO mice exhibited a larger surface area of corneal subapical nerve endings, yet fewer corneal midepithelial and subbasal nerve fibers. These findings suggest a more pronounced terminal ramification of corneal nerve endings in *Trpv1*KO mice, which could relate to the observed higher mechanosensitivity. Although Piezo2-expressing nerve endings were initially reported to terminate within the wing and basal epithelial cells and not reach the superficial squamous cell layer,^90^ a more recent study using a different method observed scarce Piezo2^+^ superficial nerve endings that also expressed TRPV1 channels.^91^ TRPV1 activation inhibits Piezo channel activity in other sensory neurons,^92^ an effect that should be absent in *Trpv1*KO mice.

Alternatively, other channel-knockout mice exhibit dysregulated gene expression in sensory neurons,^93^ which might account for the altered morphology and function reported herein. Nonetheless, the expression levels of *Piezo2* and *Trmp8* channels in the trigeminal ganglia of sham-treated *Trpv1*KO and wild-type mice were comparable (Figure 4, B and C). At any rate, these observations could serve as a starting point for further studies into corneal nerve biology.

Finally, one limitation of the present study is that despite establishing that TRPV1 facilitates DED-associated corneal neurodegeneration, it cannot ascertain in which of the many cell types found in the cornea the nonselective cation channel plays its pathogenic role. This is because in addition to sensory nerves, corneal epithelial cells, T cells, and macrophages also express TRPV1 channels that regulate their function.^68,94,95^ Regarding CD4^+^ T cells, TRPV1 channels facilitate the activation of the Th1 effector cells that contribute to corneal nerve damage.^69^ However, the potential pathogenic role of CD4^+^ T-cell–specific TRPV1 expression in the model was ruled out by adoptive transfer experiments that showed that wild-type and *Trpv1*KO CD4^+^ T cells were equally detrimental to corneal nerves. Nevertheless, the present findings do not allow us to conclude whether epithelial- or macrophage-specific TRPV1 channels drive DED-induced corneal neurodegeneration. Future studies beyond the scope of this report are needed to dissect this. At any rate, the data herein not only contribute to our understanding of how corneal nerves are affected in the setting of DED, but also indicate that corneal TRPV1 inhibition might represent a therapeutic target for corneal nerve impairment.

## Conclusions

This study demonstrates that ocular TRPV1 activation contributes to the development of DED-associated corneal nerve impairment, implying that this aspect of the disease is not merely the consequence of corneal epithelial damage but that other pathophysiological mechanisms intrinsic to corneal nerves are also involved. Ocular desiccation is sufficient to damage the most superficial nerve endings in the cornea, a process in which TRPV1 expression is not involved. However, distal-to-proximal propagation of axonal degeneration within the corneal epithelium depends on TRPV1 channels. Because both sensitization to the TRPV1 agonist capsaicin and TRPV1-dependent pain sensation take place along with disease progression, it is likely that TRPV1 overactivation is a driving force behind corneal nerve changes. On the basis of these findings, the corneal TRPV1 pathway could be a therapeutic target for corneal nerve impairment in DED and other ocular surface disorders.

## Supplementary Material

Supplemental material for this article can be found at http://doi.org/10.1016/j.ajpath.2024.01.015.

Supplemental Video S1

Supplemental Video S2

Supplementary Figure 1

Supplementary Figure 2

## Figures and Tables

**Table 1:** Reagents and Antibodies

Reagent/antibody	Product no.	Concentration/dilution	Supplier
Dextran-FITC	FD4-100 MG	10 mg/mL in PBS	Sigma-Aldrich (Buenos Aires, Argentina)
Ketamine	Ketonal 50	100 mg/kg	Richmond Vet (Grand Bourg, Argentina)
Xylazine	Xilacina 20	10 mg/kg	Richmond Vet
Capsaicin	M2028	100 μmol/L in PBS	Sigma-Aldrich
Alexa Fluor 488 anti-tubulin β3	801203	2.5–3.5 μg/μL	BioLegend (San Diego, CA)
Alexa Fluor 647 anti-mouse/human CD324 (E-cadherin)	147308	2.5 μg/μL	BioLegend
Live/Dead Fixable Dead Cell Stain	L10119	1:500 in PBS	ThermoFisher (Buenos Aires, Argentina)
CD45 allophycocyanin	103112	0.5 μL/100 μL	BioLegend
CD4 FITC	100406	0.5 μL/100 μL	BioLegend
Ly6G PE-Cy7	127618	0.5 μL/100 μL	BioLegend
IFN-γ PE	505808	0.5 μL/100 μL	BioLegend
IL-17A PE-Cy7	506922	0.5 μL/100 mL	BioLegend

Cy7: cyanine 7; FITC, fluorescein isothiocyanate; IFN-γ, interferon-γ; PBS, phosphate-buffered saline; PE, phycoerythrin.

**Table 2:** qPCR Primers

Gene name	Gene symbol Forward primer		Reverse primer	Predicted size, bp
Glyceraldehyde 3-phosphate dehydrogenase	*Gadph*	5'-CCACCACCCTGTTGC-3'	5'-CTCCCACTCTTCCACCTTCG-3'	110
Transient receptor potential cation channel subfamily V member 1	*Trpv1*	5'-CATCTTCACCACGGCTGCTTAC-3'	5'-CAGACAGGATCTCTCCAGTGAC-3'	107
Transient receptor potential cation channel subfamily M member 8	*Trpm8*	5'-GTTGGACCTTGCCAGTGATGAG-3'	5'-CCATTCTCCAGAAAGAGGCGGA-3'	129
Piezo-type mechanosensitive ion channel component 2	*Piezo2*	5'-GCACTCTACCTCAGGAAGACTG-3'	5'-CAAAGCTGTGCCACCAGGTTCT-3'	140
Activating transcription factor 3	*Atf3*	5'-GAAGATGAGAGGAAAAGGAGGCG-3'	5'-GCTCAGCATTCACACTCTCCAG-3'	120
Tumor necrosis factor	*Tnf*	5'-GGTGCCTATGTCTCAGCCTCTT-3'	5'-GCCATAGAACTGATGAGAGGGAG-3'	90

qPCR, real-time quantitative PCR.

## References

[1] Downie LE, Bandlitz S, Bergmanson JPG, Craig JP, Dutta D, Maldonado-Codina C, Ngo W, Siddireddy JS, Wolffsohn JS (2021). CLEAR - anatomy and physiology of the anterior eye. Cont Lens Anterior Eye.

[2] Galletti JG, Guzmán M, Giordano MN (2017). Mucosal immune tolerance at the ocular surface in health and disease. Immunology.

[3] Galletti JG, de Paiva CS (2021). The ocular surface immune system through the eyes of aging. Ocul Surf.

[4] Galletti JG, de Paiva CS (2021). Age-related changes in ocular mucosal tolerance: lessons learned from gut and respiratory tract immunity. Immunology.

[5] Müller LJ, Marfurt CF, Kruse F, Tervo TMT (2003). Corneal nerves: structure, contents and function. Exp Eye Res.

[6] Al-Aqaba MA, Fares U, Suleman H, Lowe J, Dua HS (2010). Architecture and distribution of human corneal nerves. Br J Ophthalmol.

[7] Vereertbrugghen A, Galletti JG (2022). Corneal nerves and their role in dry eye pathophysiology. Exp Eye Res.

[8] Craig JP, Nichols KK, Akpek EK, Caffery B, Dua HS, Joo C-K, Liu Z, Nelson JD, Nichols JJ, Tsubota K, Stapleton F (2017). TFOS DEWS II definition and classification report. Ocul Surf.

[9] Bron AJ, de Paiva CS, Chauhan SK, Bonini S, Gabison EE, Jain S, Knop E, Markoulli M, Ogawa Y, Perez V, Uchino Y (2017). TFOS DEWS II pathophysiology report. Ocul Surf.

[10] Alhatem A, Cavalcanti B, Hamrah P (2012). In vivo confocal microscopy in dry eye disease and related conditions. Semin Ophthalmol.

[11] Cruzat A, Qazi Y, Hamrah P (2017). In vivo confocal microscopy of corneal nerves in health and disease. Ocul Surf.

[12] Guerrero-Moreno A, Baudouin C, Melik Parsadaniantz S, Réaux-Le Goazigo A (2020). Morphological and functional changes of corneal nerves and their contribution to peripheral and central sensory abnormalities. Front Cell Neurosci.

[13] Labetoulle M, Baudouin C, Calonge M, Merayo-Lloves J, Boboridis KG, Akova YA, Aragona P, Geerling G, Messmer EM, Benítez-Del-Castillo J (2019). Role of corneal nerves in ocular surface homeostasis and disease. Acta Ophthalmol.

[14] Labbé A, Liang Q, Wang Z, Zhang Y, Xu L, Baudouin C, Sun X (2013). Corneal nerve structure and function in patients with non-sjogren dry eye: clinical correlations. Invest Ophthalmol Vis Sci.

[15] Tepelus TC, Chiu GB, Huang J, Huang P, Sadda SR, Irvine J, Lee OL (2017). Correlation between corneal innervation and inflammation evaluated with confocal microscopy and symptomatology in patients with dry eye syndromes: a preliminary study. Graefes Arch Clin Exp Ophthalmol.

[16] Dermer H, Hwang J, Mittal R, Cohen AK, Galor A (2022). Corneal subbasal nerve plexus microneuromas in individuals with and without dry eye. Br J Ophthalmol.

[17] Moein H-R, Akhlaq A, Dieckmann G, Abbouda A, Pondelis N, Salem Z, Müller RT, Cruzat A, Cavalcanti BM, Jamali A, Hamrah P (2020). Visualization of microneuromas by using in vivo confocal microscopy: an objective biomarker for the diagnosis of neuropathic corneal pain?. Ocul Surf.

[18] Chinnery HR, Rajan R, Jiao H, Wu M, Zhang AC, De Silva MEH, Makrai E, Stepp MA, Di Girolamo N, Downie LE (2022). Identification of presumed corneal neuromas and microneuromas using laser-scanning in vivo confocal microscopy: a systematic review. Br J Ophthalmol.

[19] Stepp MA, Pal-Ghosh S, Downie LE, Zhang AC, Chinnery HR, Machet J, Di Girolamo N (2020). Corneal epithelial “neuromas”: a case of mistaken identity?. Cornea.

[20] Simsek C, Kojima T, Dogru M, Tsubota K (2018). Alterations of murine subbasal corneal nerves after environmental dry eye stress. Invest Ophthalmol Vis Sci.

[21] Stepp MA, Pal-Ghosh S, Tadvalkar G, Williams A, Pflugfelder SC, de Paiva CS (2018). Reduced intraepithelial corneal nerve density and sensitivity accompany desiccating stress and aging in C57BL/6 mice. Exp Eye Res.

[22] Stepp MA, Pal-Ghosh S, Tadvalkar G, Williams AR, Pflugfelder SC, de Paiva CS (2018). Reduced corneal innervation in the CD25 null model of Sjögren syndrome. Int J Mol Sci.

[23] Hegarty DM, Hermes SM, Yang K, Aicher SA (2017). Select noxious stimuli induce changes on corneal nerve morphology. J Comp Neurol.

[24] Fakih D, Zhao Z, Nicolle P, Reboussin E, Joubert F, Luzu J, Labbé A, Rostène W, Baudouin C, Mélik Parsadaniantz S, Réaux-Le Goazigo A (2019). Chronic dry eye induced corneal hypersensitivity, neuroinflammatory responses, and synaptic plasticity in the mouse tri-geminal brainstem. J Neuroinflammation.

[25] Aicher SA, Hermes SM, Hegarty DM (2015). Denervation of the lacrimal gland leads to corneal hypoalgesia in a novel rat model of aqueous dry eye disease. Invest Ophthalmol Vis Sci.

[26] Belmonte C, Nichols JJ, Cox SM, Brock JA, Begley CG, Bereiter DA, Dartt DA, Galor A, Hamrah P, Ivanusic JJ, Jacobs DS (2017). TFOS DEWS II pain and sensation report. Ocul Surf.

[27] Zhang X, Volpe EA, Gandhi NB, Schaumburg CS, Siemasko KF, Pangelinan SB, Kelly SD, Hayday AC, Li D-Q, Stern ME, Niederkorn JY (2012). NK cells promote Th-17 mediated corneal barrier disruption in dry eye. PLoS One.

[28] Chen Y, Chauhan SK, Shao C, Omoto M, Inomata T, Dana R (2017). IFN-[gamma]-expressing Th17 cells are required for development of severe ocular surface autoimmunity. J Immunol.

[29] Chen Y, Chauhan SK, Lee HS, Saban DR, Dana R (2014). Chronic dry eye disease is principally mediated by effector memory Th17 cells. Mucosal Immunol.

[30] Coursey TG, Bohat R, Barbosa FL, Pflugfelder SC, de Paiva CS (2014). Desiccating stress-induced chemokine expression in the epithelium is dependent on upregulation of NKG2D/RAE-1 and release of IFN-[gamma] in experimental dry eye. J Immunol.

[31] Guzmán M, Miglio M, Keitelman I, Shiromizu CM, Sabbione F, Fuentes F, Trevani AS, Giordano MN, Galletti JG (2020). Transient tear hyperosmolarity disrupts the neuroimmune homeostasis of the ocular surface and facilitates dry eye onset. Immunology.

[32] Belmonte C, Acosta MC, Gallar J (2004). Neural basis of sensation in intact and injured corneas. Exp Eye Res.

[33] González-González O, Bech F, Gallar J, Merayo-Lloves J, Belmonte C (2017). Functional properties of sensory nerve terminals of the mouse cornea. Invest Ophthalmol Vis Sci.

[34] Schecterson LC, Pazevic AA, Yang R, Matulef K, Gordon SE (2020). TRPV1, TRPA1, and TRPM8 are expressed in axon terminals in the cornea: TRPV1 axons contain CGRP and secretogranin II; TRPA1 axons contain secretogranin 3. Mol Vis.

[35] Benítez-Angeles M, Morales-Lázaro SL, Juárez-González E, Rosenbaum T (2020). TRPV1: structure, endogenous agonists, and mechanisms. Int J Mol Sci.

[36] Mickle AD, Shepherd AJ, Mohapatra DP (2015). Sensory TRP channels: the key transducers of nociception and pain. Prog Mol Biol Transl Sci.

[37] Gouin O, L’Herondelle K, Lebonvallet N, Le Gall-Ianotto C, Sakka M, Buhé V, Plée-Gautier E, Carré J-L, Lefeuvre L, Misery L, Le Garrec R (2017). TRPV1 and TRPA1 in cutaneous neurogenic and chronic inflammation: pro-inflammatory response induced by their activation and their sensitization. Protein Cell.

[38] Guzmán M, Miglio MS, Zgajnar NR, Colado A, Almejún MB, Keitelman IA, Sabbione F, Fuentes F, Trevani AS, Giordano MN, Galletti JG (2018). The mucosal surfaces of both eyes are immunologically linked by a neurogenic inflammatory reflex involving TRPV1 and substance P. Mucosal Immunol.

[39] Shirakawa H, Yamaoka T, Sanpei K, Sasaoka H, Nakagawa T, Kaneko S (2008). TRPV1 stimulation triggers apoptotic cell death of rat cortical neurons. Biochem Biophys Res Commun.

[40] Ramírez-Barrantes R, Córdova C, Gatica S, Rodriguez B, Lozano C, Marchant I, Echeverria C, Simon F, Olivero P (2018). Transient receptor potential vanilloid 1 expression mediates capsaicin-induced cell death. Front Physiol.

[41] Maggi F, Morelli MB, Aguzzi C, Zeppa L, Nabissi M, Polidori C, Santoni G, Amantini C (2023). Calcium influx, oxidative stress, and apoptosis induced by TRPV1 in chronic myeloid leukemia cells: synergistic effects with imatinib. Front Mol Biosci.

[42] Amantini C, Mosca M, Nabissi M, Lucciarini R, Caprodossi S, Arcella A, Giangaspero F, Santoni G (2007). Capsaicin-induced apoptosis of glioma cells is mediated by TRPV1 vanilloid receptor and requires p38 MAPK activation. J Neurochem.

[43] Zhai K, Liskova A, Kubatka P, Büsselberg D (2020). Calcium entry through TRPV1: a potential target for the regulation of proliferation and apoptosis in cancerous and healthy cells. Int J Mol Sci.

[44] Li F, Yang W, Jiang H, Guo C, Huang AJW, Hu H, Liu Q (2019). TRPV1 activity and substance P release are required for corneal cold nociception. Nat Commun.

[45] Hatta A, Kurose M, Sullivan C, Okamoto K, Fujii N, Yamamura K, Meng ID (2019). Dry eye sensitizes cool cells to capsaicin-induced changes in activity via TRPV1. J Neurophysiol.

[46] Bereiter DA, Rahman M, Thompson R, Stephenson P, Saito H (2018). TRPV1 and TRPM8 channels and nocifensive behavior in a rat model for dry eye. Invest Ophthalmol Vis Sci.

[47] Fakih D, Guerrero-Moreno A, Baudouin C, Goazigo AR-L, Parsadaniantz SM (2021). Capsazepine decreases corneal pain syndrome in severe dry eye disease. J Neuroinflammation.

[48] Guzmán M, Keitelman I, Sabbione F, Trevani AS, Giordano MN, Galletti JG (2016). Desiccating stress-induced disruption of ocular surface immune tolerance drives dry eye disease. Clin Exp Immunol.

[49] Tadvalkar G, Pal-Ghosh S, Pajoohesh-Ganji A, Stepp MA (2020). The impact of euthanasia and enucleation on mouse corneal epithelial axon density and nerve terminal morphology. Ocul Surf.

[50] Schmidt U, Weigert M, Broaddus C, Myers G, Frangi AF, Schnabel JA, Davatzikos C, Alberola-López C, Fichtinger G (2018). Med Image Comput Comput Assist Interv – MICCAI 2018.

[51] Nishihara E, Hiyama TY, Noda M (2011). Osmosensitivity of transient receptor potential vanilloid 1 is synergistically enhanced by distinct activating stimuli such as temperature and protons. PLoS One.

[52] Stevenson W, Chen Y, Lee S-M, Lee HS, Hua J, Dohlman T, Shiang T, Dana R (2014). Extraorbital lacrimal gland excision: a reproducible model of severe aqueous tear-deficient dry eye disease. Cornea.

[53] Guzmán M, Keitelman I, Sabbione F, Trevani AS, Giordano MN, Galletti JG (2016). Mucosal tolerance disruption favors disease progression in an extraorbital lacrimal gland excision model of murine dry eye. Exp Eye Res.

[54] Mecum NE, Cyr D, Malon J, Demers D, Cao L, Meng ID (2019). Evaluation of corneal damage after lacrimal gland excision in male and female mice. Invest Ophthalmol Vis Sci.

[55] Parra A, Madrid R, Echevarria D, del Olmo S, Morenilla-Palao C, Acosta MC, Gallar J, Dhaka A, Viana F, Belmonte C (2010). Ocular surface wetness is regulated by TRPM8-dependent cold thermoreceptors of the cornea. Nat Med.

[56] Masuoka T, Yamashita Y, Nakano K, Takechi K, Niimura T, Tawa M, He Q, Ishizawa K, Ishibashi T (2020). Chronic tear deficiency sensitizes transient receptor potential vanilloid 1-mediated responses in corneal sensory nerves. Front Cell Neurosci.

[57] Pellegrini M, Bernabei F, Moscardelli F, Vagge A, Scotto R, Bovone C, Scorcia V, Giannaccare G (2019). Assessment of corneal fluorescein staining in different dry eye subtypes using digital image analysis. Transl Vis Sci Technol.

[58] Fabiani C, Barabino S, Rashid S, Dana MR (2009). Corneal epithelial proliferation and thickness in a mouse model of dry eye. Exp Eye Res.

[59] Beardsley RM, De Paiva CS, Power DF, Pflugfelder SC (2008). Desiccating stress decreases apical corneal epithelial cell size–modulation by the metalloproteinase inhibitor doxycycline. Cornea.

[60] Langford DJ, Bailey AL, Chanda ML, Clarke SE, Drummond TE, Echols S, Glick S, Ingrao J, Klassen-Ross T, Lacroix-Fralish ML, Matsumiya L (2010). Coding of facial expressions of pain in the laboratory mouse. Nat Methods.

[61] Mota-Rojas D, Olmos-Hernández A, Verduzco-Mendoza A, Hernández E, Martínez-Burnes J, Whittaker AL (2020). The utility of grimace scales for practical pain assessment in laboratory animals. Animals (Basel).

[62] Hunt D, Raivich G, Anderson PN (2012). Activating transcription factor 3 and the nervous system. Front Mol Neurosci.

[63] Niederkorn JY, Stern ME, Pflugfelder SC, De Paiva CS, Corrales RM, Gao J, Siemasko K (2006). Desiccating stress induces T cell-mediated Sjögren’s syndrome-like lacrimal keratoconjunctivitis. J Immunol.

[64] Chen Y, Dana R (2021). Autoimmunity in dry eye disease - an updated review of evidence on effector and memory Th17 cells in disease pathogenicity. Autoimmun Rev.

[65] Chauhan SK, El Annan J, Ecoiffier T, Goyal S, Zhang Q, Saban DR, Dana R (2009). Autoimmunity in dry eye is due to resistance of Th17 to Treg suppression. J Immunol.

[66] Foulsham W, Mittal SK, Taketani Y, Chen Y, Nakao T, Chauhan SK, Dana R (2020). Aged mice exhibit severe exacerbations of dry eye disease with an amplified memory Th17 cell response. Am J Pathol.

[67] De Paiva CS, Villarreal AL, Corrales RM, Rahman HT, Chang VY, Farley WJ, Stern ME, Niederkorn JY, Li D-Q, Pflugfelder SC (2007). Dry eye-induced conjunctival epithelial squamous metaplasia is modulated by interferon-gamma. Invest Ophthalmol Vis Sci.

[68] Bertin S, Aoki-Nonaka Y, de Jong PR, Nohara LL, Xu H, Stanwood SR, Srikanth S, Lee J, To K, Abramson L, Yu T (2014). The ion channel TRPV1 regulates the activation and proinflammatory properties of CD4^+^ T cells. Nat Immunol.

[69] Vereertbrugghen A, Pizzano M, Sabbione F, Keitelman IA, Shiromizu CM, Aguilar DV, Fuentes F, de Paiva CS, Giordano M, Trevani A, Galletti JG (2023). An ocular Th1 immune response promotes corneal nerve damage independently of the development of corneal epitheliopathy. J Neuroinflammation.

[70] Shuba YM (2020). Beyond neuronal heat sensing: diversity of TRPV1 heat-capsaicin receptor-channel functions. Front Cell Neurosci.

[71] Immke DC, Gavva NR (2006). The TRPV1 receptor and nociception. Semin Cell Dev Biol.

[72] Fakih D, Migeon T, Moreau N, Baudouin C, Goazigo AR-L, Parsadaniantz SM (2022). Transient receptor potential channels: important players in ocular pain and dry eye disease. Pharmaceutics.

[73] Morales-Lázaro SL, Simon SA, Rosenbaum T (2013). The role of endogenous molecules in modulating pain through transient receptor potential vanilloid 1 (TRPV1). J Physiol.

[74] Tominaga M, Wada M, Masu M (2001). Potentiation of capsaicin receptor activity by metabotropic ATP receptors as a possible mechanism for ATP-evoked pain and hyperalgesia. Proc Natl Acad Sci U S A.

[75] Pham TL, Bazan HEP (2021). Docosanoid signaling modulates corneal nerve regeneration: effect on tear secretion, wound healing, and neuropathic pain. J Lipid Res.

[76] Janelsins BM, Sumpter TL, Tkacheva OA, Rojas-Canales DM, Erdos G, Mathers AR, Shufesky WJ, Storkus WJ, Falo LD, Morelli AE, Larregina AT (2013). Neurokinin-1 receptor agonists bias therapeutic dendritic cells to induce type 1 immunity by licensing host dendritic cells to produce IL-12. Blood.

[77] Lighvani S, Huang X, Trivedi PP, Swanborg RH, Hazlett LD (2005). Substance P regulates natural killer cell interferon-gamma production and resistance to Pseudomonas aeruginosa infection. Eur J Immunol.

[78] Singh RB, Naderi A, Cho W, Ortiz G, Musayeva A, Dohlman TH, Chen Y, Ferrari G, Dana R (2022). Modulating the tachykinin: role of substance P and neurokinin receptor expression in ocular surface disorders. Ocul Surf.

[79] Lasagni Vitar RM, Rama P, Ferrari G (2022). The two-faced effects of nerves and neuropeptides in corneal diseases. Prog Retin Eye Res.

[80] Stepp MA, Tadvalkar G, Hakh R, Pal-Ghosh S (2017). Corneal epithelial cells function as surrogate Schwann cells for their sensory nerves. Glia.

[81] Pal-Ghosh S, Tadvalkar G, Karpinski BA, Stepp MA (2020). Diurnal control of sensory axon growth and shedding in the mouse cornea. Invest Ophthalmol Vis Sci.

[82] Goswami C, Schmidt H, Hucho F (2007). TRPV1 at nerve endings regulates growth cone morphology and movement through cytoskeleton reorganization. FEBS J.

[83] Wang S, Wang S, Asgar J, Joseph J, Ro JY, Wei F, Campbell JN, Chung M-K (2017). Ca2 and calpain mediate capsaicin-induced ablation of axonal terminals expressing transient receptor potential vanilloid 1. J Biol Chem.

[84] Wang S, Bian C, Yang J, Arora V, Gao Y, Wei F, Chung M-K (2020). Ablation of TRPV1 afferent terminals by capsaicin mediates longlasting analgesia for trigeminal neuropathic pain. eNeuro.

[85] Arora V, Li T, Kumari S, Wang S, Asgar J, Chung M-K (2022). Capsaicin-induced depolymerization of axonal microtubules mediates analgesia for trigeminal neuropathic pain. Pain.

[86] van Setten G-B (2020). Impact of attrition, intercellular shear in dry eye disease: when cells are challenged and neurons are triggered. Int J Mol Sci.

[87] Mishra SK, Tisel SM, Orestes P, Bhangoo SK, Hoon MA (2011). TRPV1-lineage neurons are required for thermal sensation. EMBO J.

[88] Mishra SK, Hoon MA (2010). Ablation of TrpV1-neurons reveals their selective role in thermal pain sensation. Mol Cell Neurosci.

[89] Cavanaugh DJ, Lee H, Lo L, Shields SD, Zylka MJ, Basbaum AI, Anderson DJ (2009). Distinct subsets of unmyelinated primary sensory fibers mediate behavioral responses to noxious thermal and mechanical stimuli. Proc Natl Acad Sci U S A.

[90] Alamri AS, Wood RJ, Ivanusic JJ, Brock JA (2018). The neurochemistry and morphology of functionally identified corneal polymodal nociceptors and cold thermoreceptors. PLoS One.

[91] Fernández-Trillo J, Florez-Paz D, íñigo-Portugués A, González-González O, Del Campo AG, González A, Viana F, Belmonte C, Gomis A (2020). Piezo2 mediates low-threshold mechanically evoked pain in the cornea. J Neurosci.

[92] Borbiro I, Badheka D, Rohacs T (2015). Activation of TRPV1 channels inhibits mechanosensitive Piezo channel activity by depleting membrane phosphoinositides. Sci Signal.

[93] Minett MS, Pereira V, Sikandar S, Matsuyama A, Lolignier S, Kanellopoulos AH, Mancini F, Iannetti GD, Bogdanov YD, Santana-Varela S, Millet Q (2015). Endogenous opioids contribute to insensitivity to pain in humans and mice lacking sodium channel Nav1.7. Nat Commun.

[94] Zhang F, Yang H, Wang Z, Mergler S, Liu H, Kawakita T, Tachado SD, Pan Z, Capó-Aponte JE, Pleyer U, Koziel H (2007). Transient receptor potential vanilloid 1 activation induces inflammatory cytokine release in corneal epithelium through MAPK signaling. J Cell Physiol.

[95] Lv Z, Xu X, Sun Z, Yang YX, Guo H, Li J, Sun K, Wu R, Xu J, Jiang Q, Ikegawa S (2021). TRPV1 alleviates osteoarthritis by inhibiting M1 macrophage polarization via Ca2+/CaMKII/Nrf2 signaling pathway. Cell Death Dis.

