# Supplementary figures and images for: Transient Receptor Potential Vanilloid-1 Channels Facilitate Axonal Degeneration of Corneal Sensory Nerves in Dry Eye
Source: Am J Pathol. Author manuscript; Available in PMC 2025 Dec 8. (PMC7618451; doi:10.1016/j.ajpath.2024.01.015)

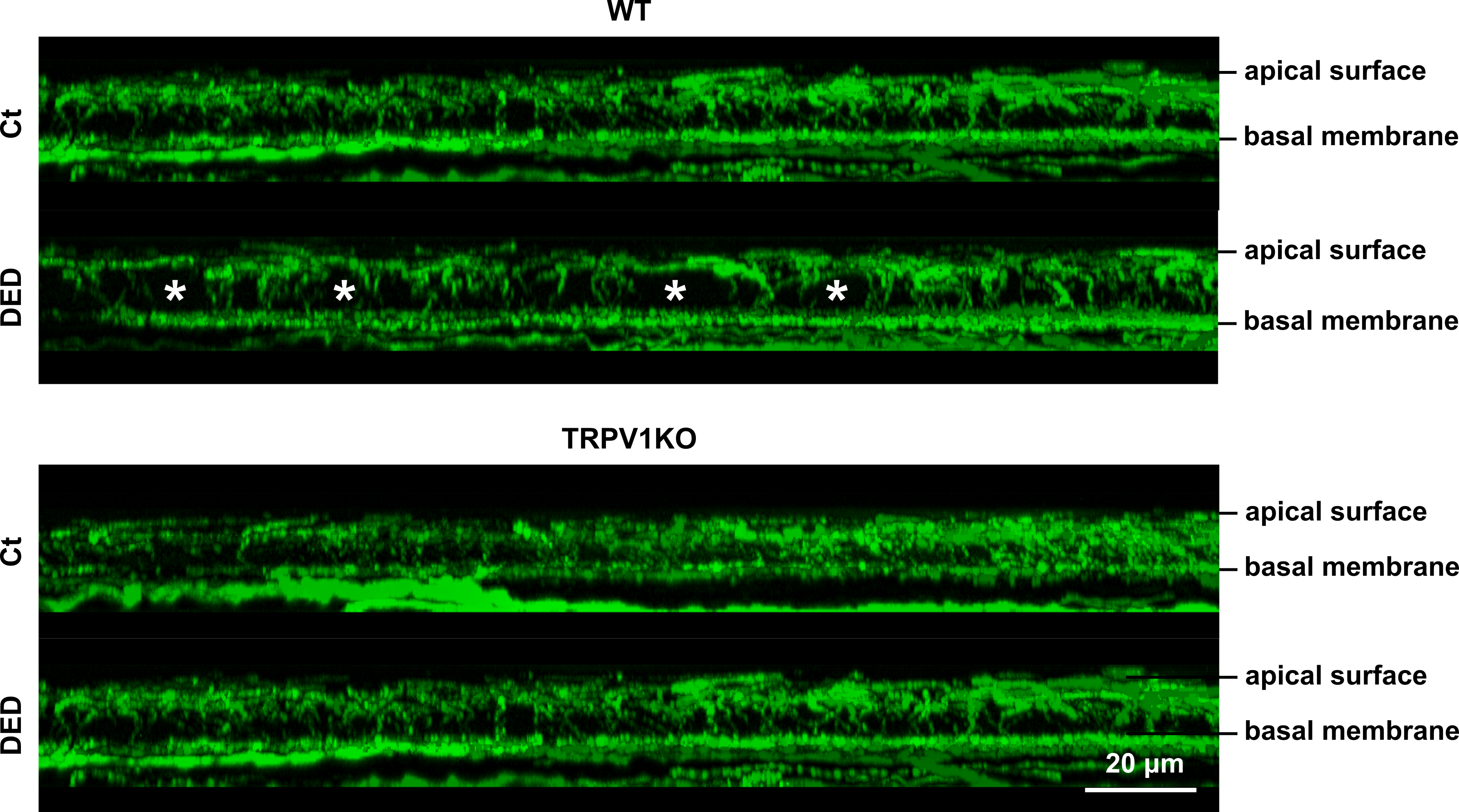

Supplement: Supplementary Figure 1 [file EMS211413-supplement-Supplementary_Figure_1.tif]

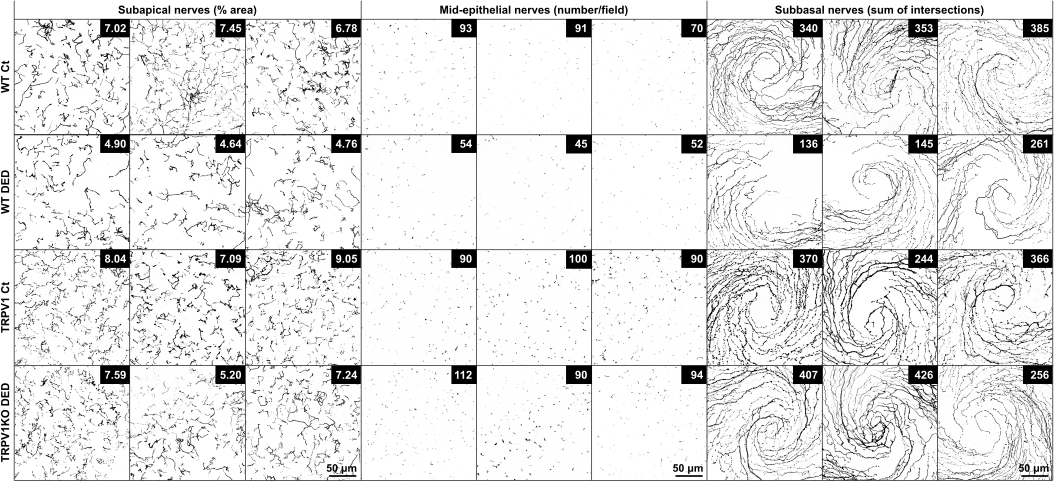

Supplement: Supplementary Figure 2 [file EMS211413-supplement-Supplementary_Figure_2.tif]
